# Transferrin Disassociates TCR from CD3 Signaling Apparatus to Promote Metastasis

**DOI:** 10.34133/research.0578

**Published:** 2025-01-13

**Authors:** Ruomei Cheng, Xiaopeng Tang, Qiyu Zhao, Yuming Wang, Wenlin Chen, Gan Wang, Chenxi Wang, James Mwangi, Qiumin Lu, Dawit Adisu Tadese, Xudong Zhao, Caiwen Ou, Ren Lai

**Affiliations:** ^1^ Key Laboratory of Genetic Evolution & Animal Models, Key Laboratory of Bioactive Peptides of Yunnan Province, KIZ-CUHK Joint Laboratory of Bioresources and Molecular Research in Common Diseases, National Resource Center for Non-Human Primates, Sino-African Joint Research Center, and New Cornerstone Science Laboratory, Kunming Institute of Zoology, The Chinese Academy of Sciences, Kunming, Yunnan 650201, China.; ^2^ Department of Clinical Laboratory, The Second Affiliated Hospital of Kunming Medical University, Kunming, Yunnan 650108, China.; ^3^ Third Department of Breast Surgery, Peking University Cancer Hospital Yunan, The Third Affiliated Hospital of Kunming Medical University, Yunnan Cancer Hospital, Kunming, Yunnan 650118, China.; ^4^ Division of Abdominal Tumor Multimodality Treatment and Laboratory of Animal Tumor Models, Cancer Center and State Key Laboratory of Respiratory Health and Multimorbidity and Frontiers Science Center for Disease-Related Molecular Network, West China Hospital, Sichuan University, Chengdu, Sichuan 610041, China.; ^5^ Department of Cardiology and Laboratory of Heart Center, Zhujiang Hospital, Guangdong Provincial Biomedical Engineering Technology Research Center for Cardiovascular Disease, Southern Medical University, Guangzhou, Guangdong 510515, China.

## Abstract

Immune recognition and activation by the peptide-laden major histocompatibility complex–T cell receptor (TCR)–CD3 complex is essential for anti-tumor immunity. Tumors may escape immune surveillance by dissembling the complex. Here, we report that transferrin, which is overexpressed in patients with liver metastasis, disassociates TCR from the CD3 signaling apparatus by targeting the constant domain (CD) of T cell receptor α (TCRα), consequently suppresses T cell activation, and inhibits anti-metastatic and anti-tumor immunity. In mouse models of melanoma and lymphoma, transferrin overexpression exacerbates liver metastasis, while its knockdown, antibody, designed peptides, and CD mutation interfering with transferrin–TCRα interaction inhibit metastasis. This work reveals a novel strategy of tumor evasion of immune surveillance by blocking the coupling between TCRs and the CD3 signaling apparatus to suppress TCR activation. Given the conservation of CD and transferrin up-regulation in metastatic tumors, the strategy might be a common metastatic mechanism. Targeting transferrin–TCRα holds promise for anti-metastatic treatment.

## Introduction

Metastasis is the primary cause of mortality in cancer patients and responsible for cancer-related deaths, rather than primary tumors [1]. The incidence of liver metastases (LMs) is 18 to 40 times higher than that of primary liver cancer, making the liver the most common site for hematogenous cancer spread [2]. The tumor cells in blood and seeding in tissues are fully exposed to immune cells. However, the molecular mechanisms of cancer cells to form metastases and evade the immune system have not been elucidated.

Growing evidence suggests that cancer or tumor cells have developed sophisticated mechanisms that hinder immune surveillance and destruction, allowing them to survive and spread throughout the body [3]. The peptide-laden major histocompatibility complex (pMHC)–T cell receptor (TCR)–CD3 complex plays a crucial role in immune recognition and activation of both in situ and metastatic tumors and has been extensively studied. For example, the major histocompatibility complex molecules, which are responsible for presenting antigens to immune cells, are decreased by cancer cells for evading immune-mediated eradication [4,5]. Secondly, cancer cells can produce immunosuppressive factors, such as cytokines and chemokines, resulting in an immunosuppressive microenvironment that dampens the immune response while promoting cancer cell survival [6,7]. Additionally, cancer cells can recruit immune suppressor cells, such as regulatory T cells [8] and myeloid-derived suppressor cells [9,10], which further suppress the immune system and facilitate tumor growth and metastasis. Moreover, cancer cells can undergo genetic mutations or epigenetic changes that result in conformational changes in the antigen, rendering them unrecognizable to the immune system and allowing them to proliferate uncontrolled [11]. Furthermore, cancer cells hijack the immune checkpoint pathway, such as programmed cell death protein 1 (PD-1) [12,13] and cytotoxic T-lymphocyte-associated protein 4 (CTLA-4) [14], which are essential for maintaining immune homeostasis and preventing excessive immune responses. Understanding these mechanisms is essential for developing novel therapies capable of overcoming immune evasion and improving cancer treatment outcomes.

In this study, we provide evidence that transferrin disrupts the formation of TCR–CD3 complexes, thereby effectively inhibiting the activation of T cells and exacerbating metastasis. Furthermore, it facilitates the up-regulation of coinhibitory molecules. Our results therefore highlight the potential of targeting the transferrin–T cell receptor α constant (TRAC) interaction as a novel LM therapeutic strategy and the vital role of the constant domain (CD) in anti-tumor immunity.

## Results

### Transferrin is up-regulated in tumor with metastases and correlates with poor outcome

**Fig. 1.**
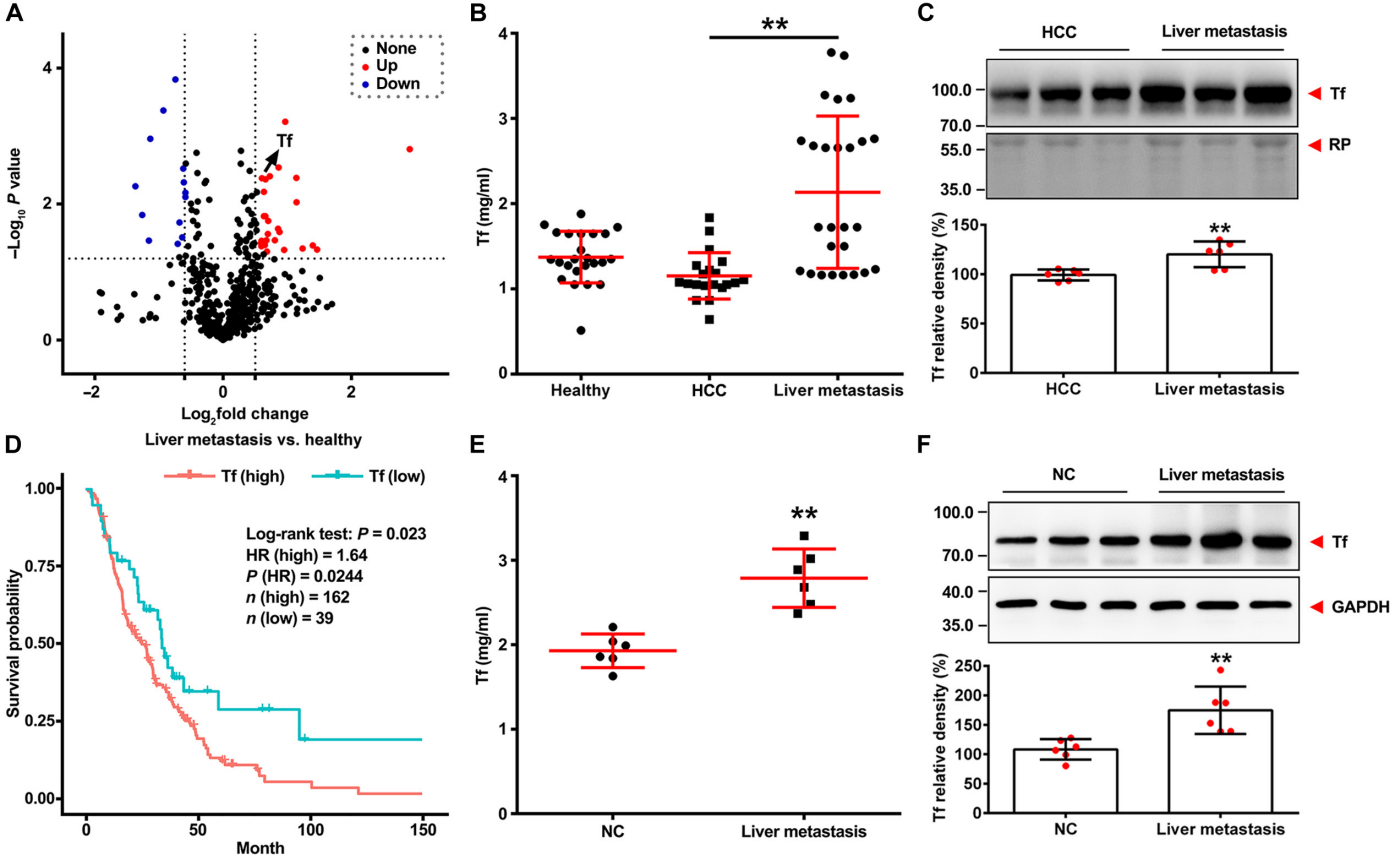
Transferrin (Tf) is elevated in liver metastases. (A) Volcano plot of proteomic protein differential analysis of plasma samples from 4 adults with liver metastases and 4 healthy individuals. Highly differentially expressed protein Tf in the plasma of individuals with liver metastases is marked by an arrow. The dataset was obtained in one experiment. (B) Enzyme-linked immunosorbent assay (ELISA) of Tf concentration in plasma from volunteers with liver metastases (*n* = 26), volunteers with hepatocellular carcinoma (HCC) (*n* = 20), and healthy volunteers (*n* = 24). Data represent mean ± SD; **P < 0.01 by unpaired *t* test. (C) Immunoblot (top) and quantification (bottom) analysis of Tf in plasma from liver metastases and HCC. Red Ponceau (RP)-stained blots were used as a loading control. Data represent mean ± SD (*n* = 6); **P < 0.01 by unpaired *t* test. (D) The association between Tf expression and the overall survival of patients with liver metastases was estimated by Kaplan–Meier analysis. *P < 0.05 by log-rank test. (E) ELISA of the Tf concentration in plasma from mice with liver metastases and negative control. Data represent mean ± SD (*n* = 6); **P < 0.01 by unpaired *t* test. (F) Immunoblot (top) and quantification (bottom) analysis of Tf in liver from liver metastases and negative control. Glyceraldehyde-3-phosphate dehydrogenase (GAPDH) was used as a loading control. Data represent mean ± SD (*n* = 6); **P < 0.01 by unpaired *t* test. HR, hazard ratio; NC, negative control.

Proteomic analysis was used to analyze differential protein expression in the plasma of individuals with LMs and healthy individuals. As illustrated in Fig. 1A, transferrin showed elevated protein expression in individuals with LM. Based on enzyme-linked immunosorbent assay (ELISA) and western blot, transferrin was up-regulated in patients of LM (Fig. 1B and C and Table S1) and the average transferrin concentration in the plasma of LM (*n* = 26) was 2.13 mg/ml (standard deviation [SD] 0.89), whereas that in hepatocellular carcinoma (HCC) (*n* = 20) was 1.15 mg/ml (SD 0.27) and healthy individuals (*n* = 24) was 1.32 mg/ml (SD 0.30) (Fig. 1B). Further, prognostic values of transferrin were analyzed based on the cBioPortal database. Statistical analysis showed that high transferrin expression levels were closely related to a low survival rate in LM patients (Fig. 1D and Table S2). We established a preclinical model of LM in melanoma by injecting B16F10 cells intrasplenically. Similarly, LM mice showed markedly elevated transferrin in both plasma and liver tissue (Fig. 1E and F). These findings suggest that transferrin expression may affect the progression and metastases of liver cancer or others.

### Transferrin promotes tumor metastasis

**Fig. 2.**
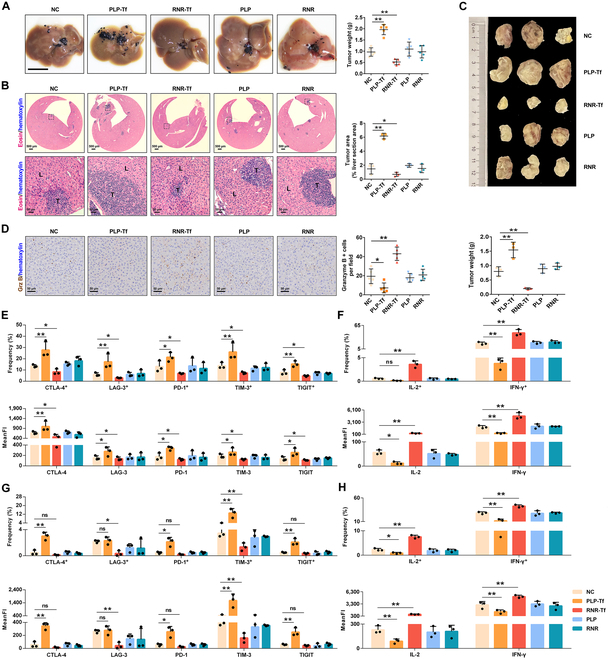
Transferrin overexpression and knockdown aggravate or attenuate liver metastasis, respectively. (A) Representative images of livers in liver metastasis mice of B16F10 tumor (left) and tumor weights (right) are shown. Scale bar, 1 cm. Images of 1 representative experiment of 5 are shown. Data represent the mean ± SD of 5 independent experiments; **P < 0.01 by one-way analysis of variance (ANOVA) with Fisher’s protected *t* test. (B) Hematoxylin-and-eosin (H&E) staining of liver sections (left) from liver metastasis mice of EL4 tumor. The tumor area is shown (right). T, tumor region; L, adjacent liver tissue. Scale bars: 500 or 50 μm. Images of 1 representative experiment of 3 are shown. (C) Representative images of tumors (top) and quantifications of tumor weights (bottom) in BALB/c mice with PLP-Tf or RNR-Tf intravenous on day 30 after subcutaneous inoculation of 1 × 10^6^ EL4 cells (*n* = 3). Data represent the mean ± SD of 3 independent experiments; *P < 0.05 and **P < 0.01 by one-way ANOVA with Fisher’s protected *t* test. (D) Immunohistochemical analysis of Granzyme B (Grz B) in liver tissues of EL4 tumor. Scale bars, 50 μm. Images of 1 representative experiment of 5 are shown. Data represent the mean ± SD of 5 independent experiments; *P < 0.05 and **P < 0.01 by one-way ANOVA with Fisher’s protected *t* test. (E) Flow cytometry analysis of the expression of cytotoxic T-lymphocyte-associated protein 4 (CTLA-4), lymphocyte activation gene 3 protein (LAG-3), programmed cell death protein 1 (PD-1), T cell immunoglobulin domain and mucin domain protein 3 (TIM-3), and T cell immunoglobulin and immunoreceptor tyrosine-based inhibitory motif domain (TIGIT) in liver metastasis mice of B16F10 tumor. Frequency and mean fluorescence intensity (MeanFI) are summarized in (E). (F) Flow cytometry analysis of the expression of interleukin-2 (IL-2) and interferon-γ (IFN-γ) in liver metastasis mice of B16F10 tumor. Frequency and MeanFI are summarized in (F). (G) Flow cytometry analysis of the expression of CTLA-4, LAG-3, PD-1, TIM-3, and TIGIT in liver metastasis mice of EL4 tumor. Frequency and MeanFI are summarized in (G). (H) Flow cytometry analysis of the expression of IL-2 and IFN-γ in liver metastasis mice of EL4 tumor. Frequency and MeanFI are summarized in (H). Data represent the mean ± SD of 3 independent experiments. ns, no significance; *P < 0.05 and **P < 0.01 by 2-way ANOVA. Tf, transferrin; PLP-Tf, Tf overexpression and its blank PLP; RNR-Tf, Tf knockdown and its blank RNR.

To further elucidate the role of transferrin in tumor metastasis, transferrin overexpression (PLP-Tf) lentiviral and knockout (RNR-Tf) retroviral were used to establish PLP-Tf and RNR-Tf mice (Fig. S1). We established 2 preclinical models of LM in melanoma (intrasplenic injection of B16F10 cells) and lymphoma (subcutaneous injection of EL4 cells) to further investigate the role of transferrin in LM. The effects of transferrin on the melanoma model were evaluated by weight of the tumors metastasized to the liver. As illustrated in Fig. 2A, transferrin overexpression significantly enhanced tumor metastasis, while knockdown of transferrin inhibited it. Similarly, the same results were also observed in lymphoma models, where transferrin overexpression significantly promoted primary tumor growth and metastasis, while transferrin knockdown inhibited primary tumor growth and metastasis (Fig. 2B and C). However, colony formation and migration assay showed that transferrin has no effect on B16F10 cell formation and migration (Fig. S2). Interestingly, immunohistochemistry analysis showed a significantly diminished level of Granzyme B in the PLP-Tf group and an elevated level in the RNR-Tf group compared to that in the control (Fig. 2D). Granzyme B is a member of the granzyme serine protease family and is found in the granules of activated cytotoxic T cells. These findings suggest that transferrin may promote tumor growth and metastasis by inhibiting T cell activity. Further, flow cytometry analysis showed that the exhaustion markers induced in LM, including CTLA-4, lymphocyte activation gene 3 protein (LAG-3), PD-1, T cell immunoglobulin domain and mucin domain protein 3 (TIM-3), and T cell immunoglobulin and immunoreceptor tyrosine-based inhibitory motif domain (TIGIT), were exacerbated by PLP-Tf but decreased following RNR-Tf in melanoma (Fig. 2E and Fig. S3A) and lymphoma (Fig. 2G and Fig. S4A). Subsequently, cytokine production (interleukin-2 [IL-2] and interferon-γ [IFN-γ]) was diminished in PLP-Tf mice, whereas it was increased in RNR-Tf mice (Fig. 2F and Fig. S3B and Fig. 2H and Fig. S4B).

### Transferrin interacts with TRAC with a high affinity and inhibits the formation of the TCR–CD3 complex

**Fig. 3.**
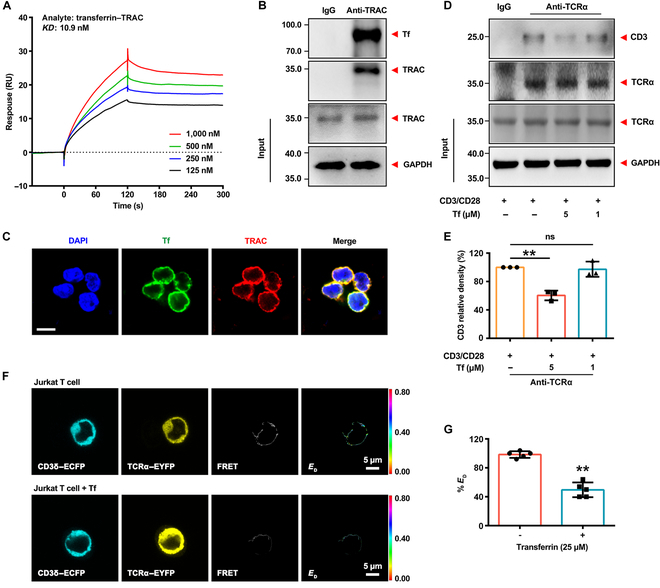
Tf interferes with the T cell receptor α chain constant (TRAC) interacting with CD3. (A) SPR analysis of the interaction of Tf with TRAC. One representative experiment of 3 is shown. (B) Coimmunoprecipitation (CoIP) and immunoblot analysis of the interaction of TRAC with Tf in human T cells. Blots of 1 representative experiment of 3 are shown. (C) Immunofluorescence analysis of the colocalization of Tf with TRAC. Scale bar, 10 μm. Images of 1 representative experiment of 3 are shown. (D and E) CoIP and immunoblot (D) and quantification (E) analysis of the intervention of Tf to TCR–CD3 interaction in human T cells. Blots of 1 representative experiment of 3 are shown. Data represent the mean ± SD of 3 independent experiments; **P < 0.01 by one-way ANOVA with Fisher’s protected *t* test. (F and G) Fluorescence resonance energy transfer (FRET) (F) and donor FRET efficiency (G) analysis of Tf interference with T cell receptor α (TCRα)–CD3δ interactions in Jurkat T cells. Scale bars, 5 μm. Images of 1 representative experiment of 5 are shown. Data represent the mean ± SD of 5 independent experiments; **P < 0.01 by one-way ANOVA with Fisher’s protected *t* test. IgG, immunoglobulin G; DAPI, 4′,6-diamidino-2-phenylindole; ECFP, enhanced cyan fluorescent protein; EYFP, enhanced yellow fluorescent protein.

The proteins that interact with transferrin were found using coimmunoprecipitation (coIP) and liquid chromatography–tandem mass spectrometry. A protein TRAC (UniProt: P01848), which is a constant region of TCRα in αβT cells, has been identified in human peripheral blood mononuclear cells (Fig. S5A and B), suggesting that transferrin may interact with αβT cells via TCRα. Interaction between transferrin with TCRα or TCRβ in vitro was examined by surface plasmon resonance (SPR) assays, suggesting that transferrin interacted with TCRα, not TCRβ (Figs. S6A and S7A and B), and the equilibrium dissociation constant (*KD*) of transferrin and recombinant TCRα was 58 nM (Fig. S6B). The TCR consists of unique variable domains (Vα and Vβ) that bind to pMHC molecules and CDs (Cα and Cβ) [15,16]. Furthermore, the interaction of TRAC with transferrin was observed (Fig. 3A to C and Fig. S7C). The interaction between transferrin and TRAC had higher affinity (*KD*: 10.9 nM) than TCRα (Fig. 3A and Fig. S7C). In human T cells, transferrin was well colocalized with TRAC (Fig. 3C), further indicating the physiological interaction of transferrin with TRAC. TCR-mediated signals are transmitted across the cell membrane by the CD3 complex. The pMHC–TCR–CD3 complex is critical for successful activation of downstream signaling pathways [17,18]. CoIP analysis showed that TCR–CD3 interactions in human T cells were interfered by transferrin (Fig. 3D and E). To monitor TCR–CD3 interactions in living cells, we generated plasmids containing CD3δ/enhanced cyan fluorescent protein (ECFP) or TCRα/enhanced yellow fluorescent protein (EYFP). CD3δ/TCRα interactions were observed by fluorescence resonance energy transfer (FRET). The efficiency of CD3δ interactions with TCRα was deduced from the donor FRET efficiency (*E*_D_). Subsequent analysis revealed that the FRET efficiency for the interaction between CD3δ and TCRα was inhibited by transferrin (25 μM) intervention (Fig. 3F and G).

### Transferrin inhibits T cell activation

**Fig. 4.**
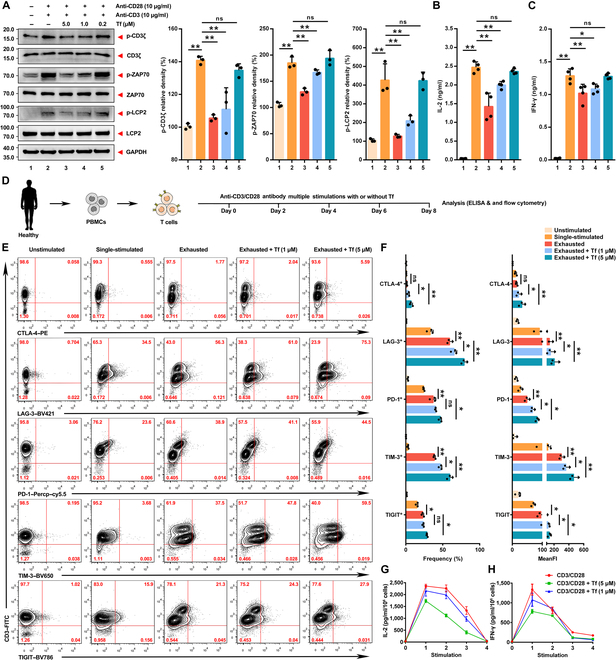
Transferrin suppresses T cell activation. (A) Immunoblot analysis of total and phosphorylated CD3ξ, zeta-chain-associated protein kinase 70 (ZAP70), and lymphocyte cytosolic protein 2 (LCP2) in human T cells. Corresponding quantifications of (A) are shown at right. GAPDH was used as loading control. Data represent the mean ± SD of 3 independent experiments. ns, no significance; **P < 0.01 by one-way ANOVA with Fisher’s protected *t* test. (B and C) ELISA of the expression of IL-2 (B) and IFN-γ (C). Isolated T cells were stimulated with anti-CD3/CD28 (10 μg/ml) for 24 h. Similar results were acquired in multiple donors (4). Data represent the mean ± SD of 4 independent experiments. ns, no significance; *P < 0.05 and **P < 0.01 by one-way ANOVA with Fisher’s protected *t* test. (D) Schematic for generation of T_EX_ cells in vitro. The model is based on the repeated stimulation of isolated T cells with anti-CD3/CD28 beads over a period of 8 d. (E and F) Flow cytometry analysis of the expression of CTLA-4, LAG-3, PD-1, TIM-3, and TIGIT in human T cells (E). As control, freshly isolated (unstimulated) or single-stimulated T cells were employed. Frequency and MeanFI are summarized in (F). Example plots are gated on viable CD3^+^ T cells. Similar results were acquired in multiple donors (3). Data represent the mean ± SD of 3 independent experiments. ns, no significance; *P < 0.05 and **P < 0.01 by 2-way ANOVA. (G and H) Isolated T cells were repeatedly stimulated with anti-CD3/CD28 beads (4 times in total). After each stimulation, the cells were counted, supernatants were collected, and the expression of IL-2 (G) and IFN-γ (H) was assessed by ELISA. Data represent the mean ± SD of 3 independent experiments. Similar data were acquired in multiple donors (3). p-CD3ξ; phosphorylated CD3ξ; p-ZAP70, phosphorylated ZAP70; p-LCP2, phosphorylated LCP2; PBMCs, peripheral blood mononuclear cells; PE, phycoerythrin; Percp, peridinin–chlorophyll–protein complex; FITC, fluorescein isothiocyanate; Tf, transferrin.

T cell signaling serves as the first signal for the activation of T cells. However, it needs to be understood that the TCR does not possess intracellular signaling domains; as a result, this prevents its direct role in T cell signaling. Instead, signaling depends on the signaling subunits of the CD3 complex to deliver information intracellularly via immunoreceptor tyrosine-based activation motifs [17,18]. Based on the extracellular contacts between TCRα and CD3δɛ interactions, it was found that they are important for normal T cell responses to TCR engagement [19,20]. As transferrin interferes with TCRα and CD3δɛ interactions, next, we evaluated if transferrin inhibited T cell signaling. Results showed that anti-CD3/CD28 (10 μg/ml) activated T cell signaling, which significantly increased the phosphorylation of CD3ξ, zeta-chain-associated protein kinase 70 (ZAP70), and lymphocyte cytosolic protein 2 (LCP2) in human T cells. Transferrin, on the other hand, significantly inhibited phosphorylation induced by anti-CD3/CD28 (Fig. 4A). Moreover, transferrin did not interfere with anti-CD3 antibody binding to CD3 (Fig. S8), which indicates that transferrin inhibits T cell activation by contacting with TCRα to inhibit the formation of the TCR–CD3 complex. T cell–derived cytokines also play a fundamental role in immunity [21–23]. Thus, the effects of transferrin on cytokine release were investigated. ELISA analysis revealed that transferrin inhibited IL-2 (Fig. 4B) and IFN-γ production (Fig. 4C) in human T cells stimulated by anti-CD3/CD28 for 24 h, confirming that T cell signaling was suppressed.

### Transferrin aggravates the expression of exhaustion markers

It is well known that TCR activation requires T cell signaling and costimulatory signals. In addition to the CD28 family of costimulatory signals, there are an increasing number of known coinhibitory molecules, such as CTLA-4, PD-1, LAG-3, TIM-3, and TIGIT [24,25]. Exhausted T cells are characterized by a gradual loss of cytokine production; coexpression of multiple coinhibitory receptors; distinct transcriptional, metabolic, and epigenetic profiles; and reduced proliferative and survival capacity [26–28]. Based on the assumption that T cell exhaustion can be attributed to persistent activation, normal freshly isolated human T cells were continuously activated with anti-CD3/CD28 over 2-d intervals to simulate a T cell exhaustion state. It has been shown previously that continuous stimulation of T cells with anti-CD3/CD28 concurrently results in the gradual down-regulation of IL-2 and IFN-γ production [29]. Similarly, repeated stimulation resulted in dramatic up-regulation of the prototypical exhaustion markers CTLA-4, LAG-3, PD-1, TIM-3, and TIGIT (Fig. 4E and F) and the loss of cytokine production ability (IL-2 and IFN-γ) concurrently (Fig. 4G and H) relative to unstimulated or single-stimulated T cells. Transferrin aggravated the expression of exhaustion markers (Fig. 4E and F) and reduced IL-2 production (Fig. 4G) compared to exhausted T cells, thus promoting T cell exhaustion. A similar pattern was observed for IFN-γ (Fig. 4H).

### Inhibition of LM in B6-*Trac*^em1^ mice

We identified 5 crucial TRAC residues (K46, R52, M54, F56, and N59) that likely play key roles in the transferrin–TRAC interaction, based on the docking model of the transferrin–TCRα complex (Fig. S9A). Mutants of TRAC (K46, R52, M54, F56, and N59) were thus constructed. Notably, the mutants R52, M54, and F56 exhibited weak interaction with transferrin (Fig. S9B). Based on the mutant results, we designed several short peptides to interfere with the transferrin–TRAC interaction. DK8 (DMRSMDFK), which is derived from the sequence of amino acids 50 to 57 in TRAC (UniProt: P01848), showed a high binding response value with transferrin to inhibit interactions of transferrin–TRAC (Fig. S9C and D) with a *KD* of 166 nM (Fig. S9E).

**Fig. 5.**
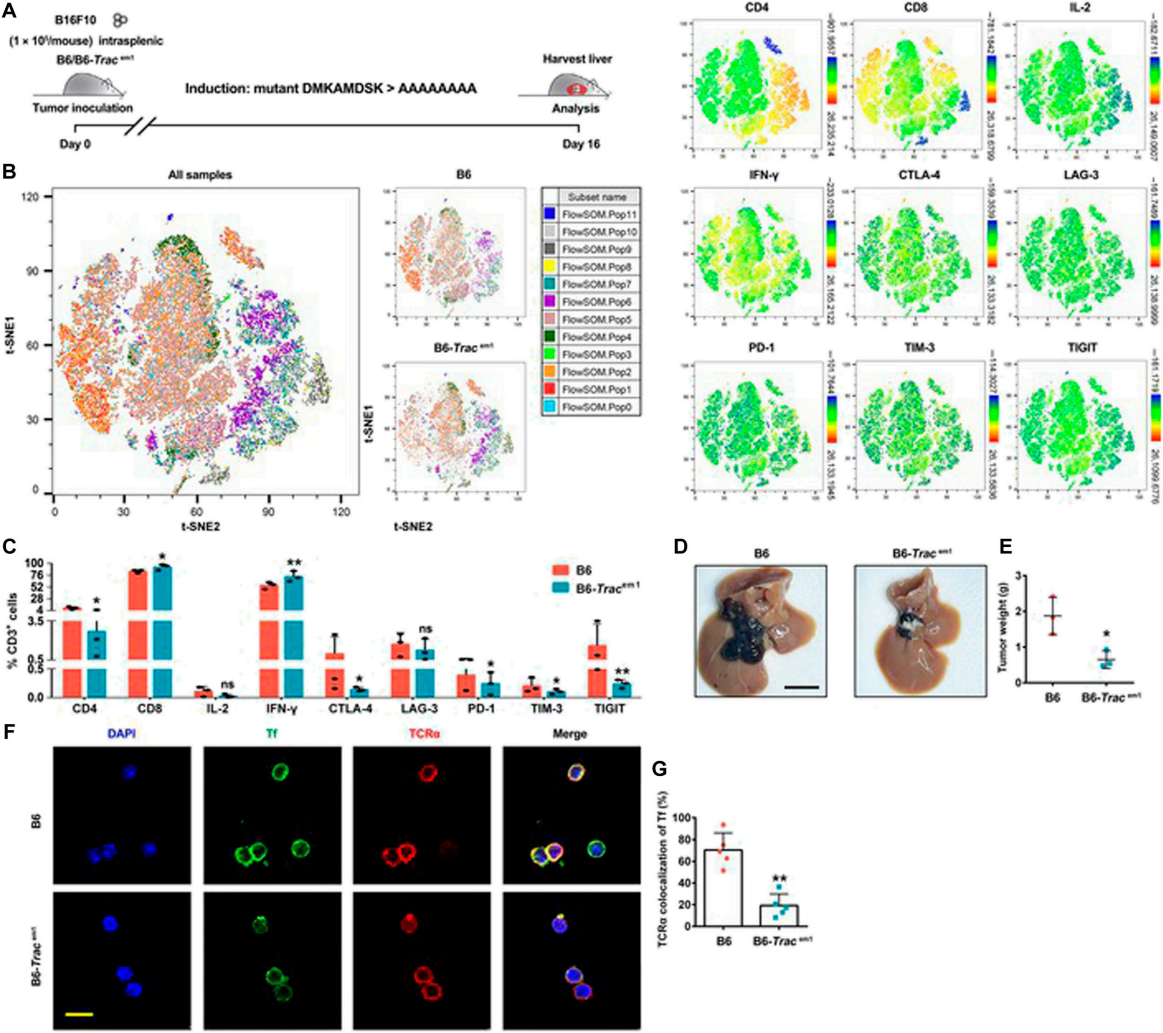
Inhibition of liver metastasis in B6-*Trac*^em1^ mice. (A) Schematic of the B6-*Trac*^em1^ mouse model of the liver metastasis of melanoma. (B and C) t-distributed stochastic neighbor embedding (t-SNE) analysis of immunophenotyping from mice (B6 or B6-*Trac*^em1^) with liver tumors. All samples combined (left, B) and single key markers (right, B) are shown. Frequency in different groups are shown in (C). Data represent the mean ± SD of 3 independent experiments. ns, no significance; *P < 0.05 and **P < 0.01 by one-way ANOVA with Fisher’s protected *t* test. (D and E) Representative images of the liver of liver metastasis of B16F10 tumor (D) and tumor weights (E) are shown. Scale bars, 1 cm. Images of 1 representative experiment of 3 are shown. Data represent the mean ± SD of 3 independent experiments, *P < 0.05 by unpaired *t* test. (F and G) Immunofluorescence (F) and quantification (G) analysis of Tf colocalization with TCRα in mice T cells. Scale bar, 10 μm. Images of 1 representative experiment of 5 are shown. Data represent the mean ± SD of 5 independent experiments; **P < 0.01 by unpaired *t* test.

We hypothesized that the DK8 mutation in TRAC could promote T cell immunity. B6-*Trac*^em1^ mice with DK8 mutation, in which DMKAMDSK (mouse-derived) was replaced with AAAAAAAA, were then generated, and an intrasplenic B16F10 tumor model was established in B6-*Trac*^em1^ mice (Fig. 5A). As illustrated in Fig. 5B and C, B6-*Trac*^em1^ mice increased the ratio of CD8^+^ T cells to CD4^+^ T cells and diminished exhaustion T cells. Moreover, B6-*Trac*^em1^ mice enhanced IFN-γ^+^ T cells in the liver. These data demonstrate that the DK8 mutant of TRAC can simultaneously blunt immunosuppression and stimulate T cell immunity. B6-*Trac*^em1^ mice showed a decreasing trend in tumor weight (Fig. 5D and E). Further results of immune colocalization showed that transferrin–TRAC interaction in T cells from B6-*Trac*^em1^ mice was reduced (Fig. 5F and G). These results indicate that the DK8 mutation in TRAC had a beneficial impact on LM inhibition.

### Therapeutic effects of transferrin antibody and interfering peptide DK8 on LM

**Fig. 6.**
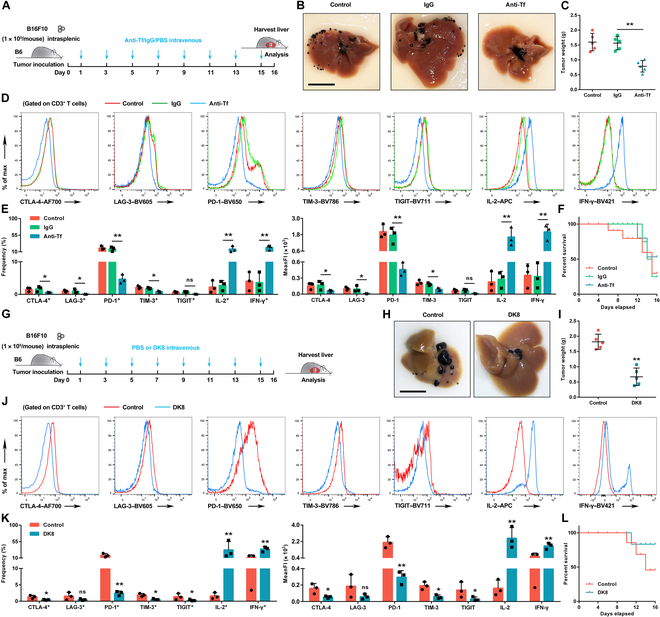
Therapeutic effects of Tf antibody and interfering peptide DK8 on the liver metastasis of melanoma. (A) Schematic of the B6 mouse model of Tf antibody intervention in the liver metastasis of melanoma. B16F10 cells (1 × 10^5^) were first intrasplenically injected into B6 mice, followed by interventions (Tf antibody [50 μg per time intravenous], isotype IgG, or phosphate-buffered saline [PBS]) every 2 d with an experimental cycle of 16 d. (B and C) Representative images of the liver of liver metastasis (B, left) and tumor weights (C, right) are shown. Mice were injected with Tf antibody (50 μg per time intravenous), isotype IgG, or PBS. Scale bar, 1 cm. Images of 1 representative experiment of 5 are shown. Data represent the mean ± SD of 5 independent experiments; **P < 0.01 by unpaired *t* test. (D and E) Histograms showing the expression levels of CTLA-4, LAG-3, PD-1, TIM-3, TIGIT, IL-2, and IFN-γ T cells in CD3^+^ T cells (D). Mice were injected with Tf antibody (50 μg per time intravenous), isotype IgG, or PBS. The frequency and MeanFI of indicated makers in each population are summarized below (E). Data represent the mean ± SD of 3 independent experiments. ns, no significance; *P < 0.05 and **P < 0.01 by one-way ANOVA with Fisher’s protected *t* test. (F) Survival curve for tumor-bearing mice from indicated groups described in (A) (*n* = 9/group). (G) Schematic of the B6 mouse model of DK8 intervention in the liver metastasis of melanoma. B16F10 cells (1 × 10^5^) were first intrasplenically injected into B6 mice, followed by interventions (DK8 [5 mg/kg intravenous] or PBS) every 2 d with an experimental cycle of 16 d. (H and I) Representative images of the liver of liver metastasis (H) and tumor weights (I) are shown. Mice were injected with DK8 (5 mg/kg intravenous) or PBS. Scale bar, 1 cm. Images of 1 representative experiment of 5 are shown. Data represent the mean ± SD of 5 independent experiments; **P < 0.01 by unpaired *t* test. (J and K) Histograms showing the expression levels of CTLA-4, LAG-3, PD-1, TIM-3, TIGIT, IL-2, and IFN-γ T cells in CD3^+^ T cells (J). Mice were injected with DK8 (5 mg/kg intravenous) or PBS. The frequency and MeanFI of indicated makers in the groups of DK8 (blue) and control (red) are summarized below (K). Data represent the mean ± SD of 3 independent experiments. ns, no significance; *P < 0.05 and **P < 0.01 by one-way ANOVA with Fisher’s protected *t* test. (L) Survival curve for tumor-bearing mice from indicated groups described in (G) (*n* = 8/group). APC, allophycocyanin.

We further assessed the therapeutic effects of polyclonal anti-transferrin antibody (anti-Tf, self-purified, 50 μg/mouse/2 d, intravenous) or DK8 (5 mg/kg/2 d, intravenous) on LM. As illustrated in Fig. 6B and C and Fig. S10B to E, administration of anti-Tf inhibited B16F10 or EL4-induced primary tumor and LM, respectively. Administration of anti-Tf also reversed increased markers of exhaustion as well as decreased cytokine production compared to immunoglobulin G (Fig. 6D and E and Fig. S10F). Moreover, in the melanoma and lymphoma models, the survival rates observed in the group treated with the antibody were 53% and 77%, respectively (Fig. 6F and Fig. S10G). In contrast, the survival rates recorded in the group administered with immunoglobulin G were 25% and 27%, while the control group of mice exhibited survival rates of 29% and 36%, at the endpoint of days 16 and 30 post tumor cell engraftment, respectively. The interfering peptide DK8 showed significant therapeutic effects by inhibiting primary tumor and LM (Fig. 6H to K and Fig. S11B to F). Moreover, tumor-bearing mice treated with DK8 demonstrated a significant remission with 83% and 62% survival in the models of melanoma and lymphoma, whereas the counterpart in phosphate-buffered saline (PBS) was only 45% (Fig. 6L) and 54% (Fig. S11G), respectively.

## Discussion

The complex interactions between cancer cells, immune cells, stromal cells, and the extracellular matrix contribute to the formation of a prometastatic niche that facilitates tumor spread [30]. The pMHC molecule is recognized by TCR on antigen-specific T cells to stimulate anti-tumor immunity. Therefore, the suppression of TCR activation by inhibiting TCR–pMHC interaction is a strategy for tumor evasion of immune surveillance. There are 3 different strategies of circulating tumor cell escape from pMHC-mediated TCR recognition, namely, interference with TCR recognition by cytokeratins, acquisition of a “pseudonormal” phenotype resulting from membrane transfer from platelets to circulating tumor cells, and major histocompatibility complex expression down-regulation or loss [3]. In this study, we identified a novel strategy that inhibits TCR activation, in which transferrin directly binds to TRAC to inhibit the formation of the TCR–CD3 signaling complex, block TCR signaling, and consequently suppress anti-metastatic and anti-tumor immunity. It also revealed the important role of the CD of TCR in anti-tumor immunity.

Proteomic analysis suggests that transferrin was elevated in the plasma of individuals with LM (Fig. 1A to C), and bioinformatic analysis suggests that high transferrin levels tightly correlate with poor survival of LM (Fig. 1D). Further mouse models showed that transferrin was elevated in both the plasma and liver tissue of LM and aggravated tumor LM, whereas LM was attenuated by transferrin knockdown (Fig. 2). Iron is an essential micronutrient required for numerous biological processes, including oxygen transport [31], energy production [32], and DNA synthesis [33]. Transferrin is responsible for maintaining the delicate balance between iron uptake, storage, and utilization via transferrin receptor (TfR) [34]. Due to increased proliferation rate and synthetic/metabolic activities in malignancy, tumor cells are more sensitive to iron. It is reported that transferrin is induced by SREBP2, a master regulator of cholesterol homeostasis, which in turn inhibits reactive oxygen species and drug-induced ferroptosis, thereby promoting tumor metastasis [35]. Moreover, elevated levels of expression of TfR have been found on cancer cells, which could be attributed to the increased need for iron [35]. Specific metabolic and molecular alterations enable disseminated tumor cells to adapt to and survive in a hypoxic microenvironment and achieve hypoxia-induced immune escape [3]. Recently, we have reported that hypoxia enhanced hypoxia-inducible factor 1α levels to promote the expression of transferrin [36–38], implying that the hypoxic microenvironment of tumors can up-regulate the transferrin level. Transferrin can be up-regulated by bacterial products like lipopolysaccharide, lipophosphocholic acid, and bacterial DNA [39]. These substances, which originate from various microorganisms, are present in many human solid tumors [40]. However, the source and function of these microorganisms remain controversial and necessitate further investigation. Granulocyte–macrophage colony-stimulating factor, which is produced primarily by tumor cells and enhances the metastatic ability of several tumors [41], induces de novo transferrin synthesis in neutrophils through the Jak/Stat5β pathway, while its neutralization curtailed neutrophil transferrin expression as well as cancer metastasis, indicating neutrophil-derived transferrin as an important regulator of metastatic tumor cell growth [42]. The up-regulation of transferrin is a complex issue that necessitates careful consideration and further in-depth research.

TCR on antigen-specific T cells recognize pMHC molecules to stimulate anti-tumor immunity [43]. TCR ligation with the pMHC, which is dependent on the variable (V) ectodomain of the αβTCR heterodimer, is central to the anti-tumor immune response. The V domain shows considerable diversity to recognize diverse antigens [44–47]. The CD of TCR receives much less attention than the V domain in anti-tumor immunity. However, increasing data indicate that CD interacts with the CD3 signaling apparatus to trigger TCR activation [48–50], implying the important role of CD in anti-tumor immunity, and the domain might be a target for evading immunosurveillance by tumors. TCR-mediated signals are transmitted across the cell membrane by the CD3 chains, leading to activation of downstream signaling pathways [17,18]. Based on the extracellular contacts between the TCRα and CD3δɛ interactions, it was found that they are important for normal T cell responses to TCR engagement [19,20]. We tested the effect of transferrin on the formation of the TCR–CD3 complex. The FRET analysis in Jurkat T cells and coIP analysis in human T cells showed that TCR–CD3 interactions were interfered by transferrin. Meanwhile, T cell signaling was significantly inhibited by transferrin (Fig. 4A to C). These data indicate that transferrin inhibited T cell activation by disrupting the association between TCR and CD3 signaling components. Mechanistically, transferrin directly interacts with TRAC with a high affinity of 10.9 nM (Fig. 3A to C) to interfere with TCR–CD3 complex interaction to suppress TCR activation and anti-tumor immunity, while blocking transferrin–TRAC interaction inhibited metastasis and showed significant anti-tumor effects. Interestingly, transferrin was found to up-regulate the expression of coinhibitory molecules (Fig. 4E and F). To date, a number of key transcription factors were identified that control the degree of expression of coinhibitory molecules [51–55]. Therefore, more systematic studies remain to address the mechanism. Immunotherapies like chimeric antigen receptor T cells and bispecific antibodies offer promising strategies for treating tumors by redirecting T cells against cancer cells, and these approaches can face primary resistance and acquired resistance. Studies have shown that the abundance of exhausted CD8^+^ T cell clones is associated with resistance to BCMA×CD3 bispecific T cell engagers in patients with multiple myeloma [56]. Consistently, several baseline immune characteristics that predict a poor response to this T cell engager include reduced T cell counts; increased levels of T cells expressing PD-1 and TIM-3; and a decreased proportion of naive T cells [57]. The data indicate that transferrin increased the expression of baseline immune characteristics associated with poor response to immunotherapy. However, whether transferrin actually promotes resistance to immunotherapy requires further investigation.

Previous studies have indicated that TfR might be a potential therapeutic target for cancer therapy [58]. However, the clinical application of this concept has been limited due to concerns about the potential for severe toxicity if the receptor is targeted systemically, given its critical role in binding to transferrin for iron transport to normal cells [34,58]. In humans, transferrin is normally about 30% saturated with iron [59]. Hence, the use of anti-transferrin antibodies to counteract the up-regulation of transferrin could offer a potential novel therapeutic approach. The current finding also provides a promising alternative by selectively blocking the interaction between transferrin and TRAC without directly targeting TfR to affect iron transport. Here, we demonstrated that polyclonal anti-transferrin antibody (anti-Tf, self-purified) or a designed peptide (DK8) that selectively blocked the transferrin–TRAC interaction neutralized the immunosuppression of T cells caused by up-regulated transferrin. This led to significantly reduced metastasis in the mouse models of melanoma and lymphoma, making it a potential target for cancer therapy without interfering with its homeostatic functions. Collectively, the CD of TCR is found to be an important target for anti-tumor treatment, and its interaction with transferrin may have immune-checkpoint-like functions.

Our study has limitations. We note that the currently available preclinical models of LMs that we used may not fully represent human pathophysiology. However, the currently available humanized models [60] have the limitation of human leukocyte antigen mismatch between the tumor and human immune cells, which may induce treatment-independent immune response to tumor cells [61]. If advanced humanized mouse models become available in the future, we will conduct necessary research to further evaluate anti-Tf or the designed peptide (DK8).

## Materials and Methods

### Human subjects

For comparison of transferrin concentrations, 24 plasma samples from healthy controls, 20 plasma samples from patients confirmed with HCC, and 26 plasma samples from patients confirmed with tumor LM were collected from the Second Affiliated Hospital of Kunming Medical University and the Third Affiliated Hospital of Kunming Medical University (Table S1). This study was approved by the Institutional Review Board of the Kunming Institute of Zoology (approval IDs: SMKX-20180724-166 and KIZRKX-2024-002). All specimens were collected with patients’ informed consent prior to the study. The collected plasma samples were stored at −80 °C pending analysis.

### Mice

Eight- to 10-week-old female C57BL/6J (referred as B6 mice) and BALB/c mice were ordered (Shanghai Model Organisms Center, Inc., China). The B6-*Trac*^em1^ mice were generated by using the CRISPR/Cas9 system to recombine the DMKAMDSK knock-in mutation of B6 background mice and validated by sequencing with primers (forward primer: 5′-CAGAAAGGACCCCGTGGAGAGG-3′; reverse primer: 5′-ATGGGCAGGAAGGGAATGGAAACT-3′) (Shanghai Model Organisms Center, Inc., China). All mice were maintained under specific pathogen-free housing with a maximum of 5 mice per cage. All animal experiments performed in this study were approved by the Life Science Ethics Committee of the Kunming Institute of Zoology, Chinese Academy of Sciences (approval ID: SMKX-20180724-166). All possible efforts were made to minimize animal suffering.

### Cell lines

B16F10 (Cat.: KCB2014047YJ) and EL4 tumor cells (Cat.: KCB2013027YJ) were acquired, identified, and authenticated by the Conservation Genetics CAS Kunming Cell Bank. B16F10 was cultured in Dulbecco’s modified Eagle medium/nutrient mixture F-12 (10-092-CVR, Corning, USA) containing 10% fetal bovine serum (35-081-CV, Corning, USA) and 1% penicillin–streptomycin solution (P1400, Solarbio, China). EL4 was cultured in RPMI 1640 medium (10-040-CVR, Corning, USA) supplemented with 10% horse serum (S9050, Solarbio, China) and 1% penicillin–streptomycin solution. The mouse normal embryonic liver cell line (BNL CL.2; Cat.: KCB2012114YJ) and Jurkat T cell line (Cat.: KCB 94008YJ) were also obtained from the Cell Bank. BNL CL.2 cells were maintained in Dulbecco’s modified Eagle medium (10-013-CVR, Corning, USA), and Jurkat T cells were maintained in RPMI 1640 medium containing 10% fetal bovine serum and 1% penicillin–streptomycin solution. The cells were cultured at 37 °C in 5% CO_2_, 95% air-humidified incubators.

### SPR analysis

A Biacore T200 instrument (GE, USA) and a CM5 sensor chip (29149603, Cytiva, USA) were employed. To analyze the interaction between transferrin with TCRα and TCRβ, the CM5 sensor chip was first activated by 100 μl of *N*-hydroxysuccinimide and 1-ethyl-3-[3-dimethylaminopropyl]carbodiimide hydrochloride at a flow rate of 5 μl/min. Transferrin diluted (20 μg/ml) with 200 μl of sodium acetate (10 mM, pH 4.5) was then captured on the CM5 sensor chip at ~6,000 resonance units. The remaining activated sites on the CM5 sensor chip were blocked by 100 μl of ethanolamine (1 M, pH 8.5) at a flow rate of 5 μl/min. Proteins of TCRα, TCRβ, and human serum albumin (1,000 nM) in PBS with Tween 20 (PBST) buffer (02-024-1ACS, Biological Industries, Israel, with 0.02% Tween 20 [v/v)]) were applied to analyze interactions with transferrin on the surface of the CM5 sensor chip at a flow rate of 20 μl/min. Different concentrations of TCRα and TRAC (125, 250, 500, and 1,000 nM) in PBST buffer were then analyzed. The equilibrium dissociation constant (*KD*) for binding, as well as the association (*Ka*) and dissociation (*Kd*) rate constants, were fit to kinetic and steady-state models using Biacore T200 Evaluation Software v3.0. The effects of transferrin on anti-CD3 antibody–CD3 interaction were also investigated by SPR. Anti-CD3 antibody (10 μg/ml, 130-093-387, Miltenyi, Germany) was biotinylated according to the manufacturer’s protocols. Biotinylated anti-CD3 antibody was dissolved in PBST buffer and coupled with a streptavidin (SA) sensor chip (29104992, Cytiva, USA). CD3 (100 nM) was then flowed across the SA sensor chip to combine with the immobilized anti-CD3 antibody. CD3 (100 nM) mixed with different concentrations of transferrin (100 and 500 nM) was applied to analyze the blockage of transferrin on anti-CD3 antibody–CD3 interaction at a flow rate of 20 μl/min.

### FRET imaging by sensitized emission

We generated plasmids containing CD3δ/ECFP or TCRα/EYFP (VectorBuilder, China). ECFP attached to the N-terminus of CD3δ (UniProt: P04234), and EYFP linked to the N-terminus of TCRα (UniProt: P0DTU3). The Jurkat T cells transfectants were prepared according to the Lipofectamine 3000 reagent protocol (L3000001, Invitrogen, USA) and grown for 24 h before imaging. Prior to FRET, the culture medium was changed for serum-free medium under 5% CO_2_, and a human CD3/CD28/CD2 T cell activator was added with or without transferrin (25 μM) intervention. The glass bottom dishes were analyzed in a heated tissue culture chamber at 37 °C.

FRET between ECFP and EYFP molecules was studied by calculating the sensitized emission (the EYFP emission upon ECFP excitation) from separately acquired donor and acceptor images. Images were acquired on a Zeiss LSM880 confocal microscope (Germany). Three images were collected: ECFP excited at 458 nm and detected between 470 and 500 nm, indirect EYFP excited at 458 nm and detected between 520 and 560 nm, and direct EYFP excited at 514 nm and detected between 520 and 560 nm [62]. Because of considerable overlap of ECFP and EYFP spectra, EYFP emission was corrected for leakthrough of ECFP emission and for direct excitation of EYFP during ECFP excitation.

FRET was calculated from these data as described in detail [63]. In brief, the images were shade-corrected and optionally smoothed. Sensitized emission (*F*_Sen_) was calculated using correction factors obtained from cells expressing either ECFP or EYFP alone, which were updated for every image. Then, the apparent FRET efficiency was calculated by relating *F*_Sen_ to the total donor level (*E*_D_) using the ImageJ plug-in PixFRET [64].

### Transferrin inhibits T cell signaling stimulated by anti-CD3/CD28

T cells were seeded into 24-well plates at 1 × 10^6^ cells/well and incubated with 10 μg/ml anti-CD3 antibody (130-093-387, Miltenyi, Germany), 10 μg/ml anti-CD28 antibody (130-093-375, Miltenyi, Germany), and transferrin (0.2, 1, and 5 μM) in medium for 30 min or 24 h, respectively. Western blot analysis was used to test the phosphorylation of the CD3ξ, ZAP70, and LCP2 subunits of the T cell signaling pathway. The cell supernatant IL-2 and IFN-γ levels were also tested using the ELISA kit.

### Transferrin aggravates the expression of exhaustion markers

The generation of human exhausted T cells in vitro has been described in previous work [29]. In brief, isolated T cells were seeded into 24-well plates at 1 × 10^6^ cells/well, and repeated stimulation was performed with 10 μg/ml anti-CD3 antibody and 10 μg/ml anti-CD28 antibody with or without transferrin (1 and 5 μM) over 2-d intervals (Fig. 2G). As control, freshly isolated (unstimulated) or single-stimulated T cells were employed. Over a period of 8 d, flow cytometry analysis was used to test the expression of CTLA-4, LAG-3, PD-1, TIM-3, and TIGIT. After each stimulation, the cells were counted, supernatants were collected, and the expressions of IL-2 and IFN-γ were assessed by ELISA.

### Tumor models

For experimental LM models, melanoma (B16F10 cells, 1 × 10^5^) and lymphoma (EL4, 1 × 10^6^) were intrasplenically or subcutaneously injected as described [65,66]. Mice were sacrificed once the tumor burden reached 2,000 mm^3^. For caliper measurement, tumor volume was calculated using the formula (*π*/6) × (*L* × *W*^2^), where *L* represents length and *W* represents width. The time points for the experimental LM models of melanoma and lymphoma were 16 and 30 d, respectively. The weight of the excised tumor was determined in the mouse models of melanoma, and the tumor area was calculated according to hematoxylin-and-eosin staining of liver sections in the mouse models of lymphoma using ImageJ.

### Preparation of single-cell suspensions from liver

Mouse livers were removed and immediately placed in PBS buffer. The excised livers were sliced into small pieces and filtrated through a 70-μm sterilized cell strainer. The collected suspension was washed twice with PBS buffer at 1,000 rpm after lysing red blood cells. Cell pellets were resuspended in 33% Percoll (P8370, Solarbio, China) and separated by centrifugation in a discontinuous Percoll gradient (33%/70%) at 2,400 rpm for 30 min with a speed reduction setting to 1 at room temperature. Lymphocytes were isolated from the interphase, washed, and stained for flow cytometry.

### Flow cytometry

Human T cells and mice mononuclear cells were stained with fluorescently conjugated antibodies. Prior to surface antigen staining, dead cells were stained by Fixable Viability Stain 780 (565388, BD Biosciences, USA) for 30 min at 4 °C. Extracellular staining using the antibodies listed below was performed for 30 min at 4 °C, and then the cells were washed and resuspended in 0.5 ml of freshly prepared Fix/Perm solution (88-8824-00, Invitrogen, USA) at 4 °C overnight. After being washed with Perm/Wash buffer (88-8824-00, Invitrogen, USA), the cells were stained with intracellular antibodies listed below. Data collection was performed using BD LSRFortessa equipped with 5 lasers (BD Biosciences), and the data were analyzed with the BD FlowJo software (v10.8.1). The following antibodies were used: CD3 (clone UCHT1, 300406, BioLegend; clone 145-2C11, 553061, BD Biosciences), CD8 (clone 53-6.7, 100752, BioLegend), CD4 (clone GK1.5, 100410, BioLegend), IL-2 (clone MQ1-17H12, 566405, BD Biosciences; clone JES6-5H4, 554429, BD Biosciences), IFN-γ (clone B27, 562974, BD Biosciences; clone XMG1.2, 563376, BD Biosciences), CTLA-4 (clone BNI3, 555853, BD Biosciences; clone UC10-4B9, 106323, BioLegend), LAG-3 (clone 3DS223H, 48-2239-42, eBioscience; clone C9B7W, 125227, BioLegend), PD-1 (clone MIH4, 46-9969-42, eBioscience; clone 29F.1A12, 135231, BioLegend), TIM-3 (clone F38-2E2, 25-3109-42, eBioscience; clone RMT3-23, 119725, BioLegend), and TIGIT (clone 741182, 747838, BD Biosciences; clone 1G9, 565168, BD Biosciences). All antibodies were used at a 1:100 dilution.

### Statistical analysis

The data obtained from independent experiments are presented as mean ± SD. Results were analyzed using unpaired *t* test or one-way analysis of variance (ANOVA) with Fisher’s protected *t* tests in Prism 6 (GraphPad Software) and SPSS v22.0 (SPSS Inc., USA). Differences were considered significant at *P* < 0.05.

## Data Availability

The data that support the findings of this study are available from the corresponding authors upon reasonable request.

## Supplementary Materials

Supplementary 1 Supplementary Methods Figs. S1 to S12 Tables S1 and S2 Supplementary Reference
