# Supplementary material for: Transferrin Disassociates TCR from CD3 Signaling Apparatus to Promote Metastasis
Source: Research (Wash D C). 2025 Jan 13;8:0578. doi: 10.34133/research.0578 (PMC11731779; doi:10.34133/research.0578)
Supplement: Supplementary 1 — Supplementary Methods Figs. S1 to S12 Tables S1 and S2 Supplementary Reference [file research.0578.f1.zip › Revised Supplemental Materials.docx]

**Supplemental Materials include:**

**1. Supplementary Methods**

**2. Supplementary Figures**

**3. Supplementary Tables**

**4. Supplementary References**

**Supplementary Methods**

**Isolation of human peripheral blood mononuclear cells (PBMCs) and T cells**

Healthy human peripheral blood was treated with a 1.5% EDTA-Na_2_ anticoagulant agent. The PBMCs were isolated by density gradient centrifugation using Polymorphprep (AS1114683, Axis-Shield, Norway) according to the manufacturer’s protocols. In brief, 5 mL of anticoagulated peripheral blood was carefully layered over 5 mL of Polymorphprep, and the samples were then centrifuged at 500 ×*g* for 30 min with speed reduction setting to 2 in a swing-out rotor at 22 °C. After centrifugation, two leucocyte bands were visible and the top leucocyte band consisted of PBMCs. The top band was harvested and diluted with five volumes of red blood cell lysis buffer (J2219, Warbio, China). The remaining red blood cells were then removed by centrifugation at 500 ×*g* for 10 min at 22 °C. The collected cells were washed with three volumes of PBS buffer (02-024-1ACS, Biological Industries, Israel) and centrifuged at 120 ×*g* for 10 min to remove platelets. The isolated PBMCs were used for subsequent experiments. To isolate T cells, human T cell isolation kit (17951, Stemcell, Canada) was used by immunomagnetic negative selection according to the manufacturer’s protocols. Isolated T cells were cultured in T cell expansion medium (10981, Stemcell, Canada) with human CD3/CD28/CD2 T Cell activator (25 μL/10^6^ cells, 10990, Stemcell, Canada) and IL-2 (10 ng/mL, AF-200-02-100, Peprotech, USA).

**Transferrin binding protein identification from human PBMCs**

Human PBMCs (2 × 10^7^ cells) was lysed in ice-cold NP-40 buffer (0.025 mM Tris-HCl, pH7.4, 0.015 mM NaCl, 1% NP-40, 5% glycerol, 0.001 mM EDTA-Na_2_) for 30 min with protease inhibitors (HY-K0010, MedChem Express, USA) and phosphatase inhibitors (HY-K0022, MedChem Express, USA). Dynabeads Protein A (10002D, ThermoFisher Scientific, USA) was then added and mixed with anti-transferrin antibody (1:500, 11019-RP02, Sino Biological, USA) or control immunoglobulin G (IgG, NI01, Sigma, USA) with transferrin (5 μM, T2036, Sigma, USA) for 4 h at 4 ℃. The beads were washed with PBS three times. The coupling proteins were then eluted by glycine (2 M, pH 2.0). All proteins undergo enzymatic digestion and liquid chromatography-tandem mass spectrometry analysis (LC-MS, Ultimate 3000 nano-LC-Orbitrap Q Exactive Mass Spectrometer, ThermoFisher Scientific, USA). The database retrieval algorithm used was Percolator. The database used for searching was the UniProt proteome reference database for humans.

**Expression and purification of human TCRα, TCRβ and TRAC recombinant protein**

TCRα, TCRβ and T cell receptor α constant (TRAC) were expressed using the Expi293^TM^ Expression Systems (A14635, ThermoFisher, USA). The plasmids for TCRα, TCRβ and TRAC were transfected in human embryonic kidney 293F (HEK293F) cells. The HEK293F cells were maintained in SMM 293-TI Expression Medium (M293TI, Sino Biological, China). The cells were cultured at 37 ℃ in 5% CO_2_, 95% air humidified incubators. Culture medium was collected on the fifth day post transfection. The supernatant containing the TCRα, TCRβ or TRAC recombinant protein was passed through a Ni^2+^ affinity chromatography column (30230, QIAGEN, Germany) or MabSelect SuRe LX column (29340185, GE, USA) to obtain pure protein. Recombinant TCRα and TCRβ protein were then subjected to 12% sodium dodecyl sulfate-polyacrylamide gel electrophoresis (SDS-PAGE) and immunoblotting for purity analysis. Recombinant TRAC was obtained by TEV protease digestion, followed by MabSelect SuRe LX column purification. Purity analysis was conducted using immunoblotting. The recombinant TCRα, TCRβ and TRAC proteins were used for subsequent experiments.

**Colony formation assay**

Colony formation was performed by seeding in 6-well plates and incubated with transferrin (1, 5 μM) for 24 h. One week after treatment, the cells were fixed in ice-cold methanol for 10 min and stained with 1% crystal violet (G1062, Solarbio, China) for 15 min. The colonies were rinsed with water and counted using Image J. The experiment was carried out in triplicates.

**Cell migration assay**

The migration potentials of the cells were evaluated using transwell inserts with pores of 8 μm (3422, Corning, USA). 24 h after transfection, 3.0 × 10^5^ cells in serum-free medium were added to the upper insert pre-coated with matrigel matrix. 700 μL 10% FBS medium was added to the lower chamber. After incubation for 24 h, non-invading cells were removed from the upper surface of the transwell membrane with a cotton swab and the invaded cells on the lower membrane surface were fixed in methanol, stained with 0.1% crystal violet, photographed, and then counted. Cells in five random fields at 10 × magnification for each insert were counted. The assays were conducted in triplicate.

**Co-immunoprecipitation (Co-IP) and immunoblot analysis of protein-protein interactions**

To analyze the interaction of TRAC with transferrin *in* *vitro*, T cells were lysed in ice-cold NP-40 buffer for 30 min with protease inhibitor. Protein A/G beads (88803, Thermo Fisher, USA) were used according to the manufacturer’s instructions. Briefly, protein A/G beads were washed with PBS and co-incubated with cell lysates in the presence of TCRα antibody (sc-515719, Santa, USA) or control immunoglobulin (IgG, ab198772, Abcam, USA) for 4 h at 4 ℃. The beads were washed with PBS three times. The coupling proteins were then eluted by boiling the beads in 1 × sodium dodecyl sulphate (SDS) for 10 min. To assay inhibition of transferrin on the interaction of TCRα with CD3, T cells were activated with human CD3/CD28/CD2 T cell activator for 30 min. The T cells were lysed in ice-cold NP-40 buffer and co-incubated with protein A/G beads and TCRα antibody (sc-515719, Santa, USA) with or without transferrin (1, 5 μM, T2036, Sigma, USA) or IgG (ab198772, Abcam, USA) overnight at 4 ℃. All proteins were separated by 12% SDS-PAGE, transferred onto polyvinylidene ﬂuoride (PVDF) membranes, blocked with 5% bovine serum albumin (BSA) for 2 h at room temperature, immunoblotted with anti-Tf antibody (1:1000, ab277635, Abcam, USA), anti-TCRα antibody (1:500, sc-515719, Santa, USA), anti-CD3ԑ antibody (1:5000, ab133628, Abcam, USA), and anti-GAPDH antibody (1:2000, T0004, Affinity, USA).

**Immunofluorescence analysis**

To analyze confocal co-localization of transferrin with TRAC, human T cells were activated with human CD3/CD28/CD2 T cell activator for 30 min, fixed in 4% paraformaldehyde for 30 min, permeabilized with 0.2% Triton X-100 and blocked with 2% bovine serum albumin (BSA) for 1 h. The cells were incubated with primary antibodies (anti-Tf antibody (1:500, ab277635, Abcam, USA) and anti-TCRα antibody (1:100, sc-515719, Santa, USA)) overnight at 4 ℃. After washing with PBS, the cells were incubated with fluorescent secondary antibodies (1:400; FITC anti-Rabbit IgG, 111-095-144, Jackson, USA; or Cy3 anti-Mouse IgG, 715-165-151, Jackson, USA) at room temperature for 1 h in the dark, washed thoroughly with PBS and sealed with ProLong Gold Antifade Reagent with DAPI (8961S, CST, USA). Immunostaining was detected using an Olympus FluoView 1000 Confocal Microscope (Japan). Images were analyzed using Image J.

**Generation of lentiviral and retroviral vectors and virus package for transferrin overexpression and knockdown**

Transferrin overexpression and knockdown vectors were constructed. HEK 293T (Conservation Genetics CAS Kunming Cell Bank, China) and EcoPack™ 2-293 cells (Clontech, USA) were used for packaging lentiviruses and retroviruses, respectively, as our previous work[1].

**Polyclonal anti-transferrin antibody preparation**

The anti-transferrin polyclonal antibody was produced as the method described in previous work[1]. In brief, mouse transferrin (500 μg, T0523, Sigma, USA) in 300 μL of 0.9% NaCl was mixed with 300 μL of Freund's Complete Adjuvant (Sigma, USA) and injected subcutaneously into the backs of New Zealand white rabbits (male, 3 months old, 2 kg) on day 0. The mixture of antigen and Freund's Incomplete Adjuvant (Sigma, USA) was used for second (day 14) and third (day 28) immunizations. Ten days following the third immunization, blood samples were collected from the rabbit and assessed for valence using the enzyme-linked immunosorbent assay (ELISA). Anti-transferrin polyclonal antibodies were then purified from the rabbit serum on a protein A column (17040303, Cytiva, USA).

**Protein-protein docking**

To model the transferrin-TCRα complex, we used the known structure of transferrin and TCRα for protein docking. The crystal structure of transferrin (PDB ID: 3QYT) was docked to the structure of TCRα (PDB ID: 1OGA) by ZDOCK. In predicting protein-protein complexes, ZDOCK considers shape complementarity, electrostatics, and desolvation free energy (https://doi.org/10.1002/prot.10389). About 2 000 structure complexes were generated and ranked according to the ZRANK scoring function. The best ZDOCK pose between the two conformations was used as a representative of transferrin-TCRα interaction.

**Hematoxylin-and-eosin (H&E) staining**

The formalin-fixed, paraffin-embedded liver tissues were cut into 4-μm slices, dewaxed, stained with hematoxylin solution (G1005-1, Solarbio, China) for 5 min at room temperature and then rinsed in tap water for 10 min. Next, 1% ethanol hydrochloride in water was used to diferentiate the sample for 30 s, followed by rinsing with tap water. Finally, staining was performed with 0.5% eosin solution (G1005-2, Solarbio, China) for 5 min and rinsed in tap water for 30 s. Images were captured using a slide scanning image system (Shenzhen Shengqiang Technology Co., Ltd, China) and analyzed using Image J.

**Immunohistochemistry analysis**

Immunohistochemistry (IHC) was performed on 5 μm sections of formalin fixed using antibodies targeting the Granzyme B (1:500, 13-8822-82, Thermo Fischer, USA). Before antibody incubation, sections of tissues were subjected to 15 min of antigen retrieval (10 mM citrate buffer, pH 6.0, 98 °C). A secondary antibody conjugated to a horseradish peroxidase (HRP)-labeled (1:500, 112-035-003, Jackson ImmunoResearch, USA) was used. The targeting were quantified using ImageJ software.

**Immunoblot analysis**

Total proteins were extracted by RIPA Buffer (R0278, Sigma-Aldrich, USA) containing protease inhibitors and phosphatase inhibitors. Samples were separated by 12% SDS-PAGE and further transferred to PVDF membranes. The membranes were blocked with 5% BSA dissolved in [TBST](http://www.thelabrat.com/protocols/TBST.shtml) buffer (2.42 g/L Tris base, 8 g/L NaCl, 0.1% Tween-20 (v/v), pH 7.6) for 2 h at room temperature. After washing three times with TBST buffer, the PVDF membranes were incubated overnight in primary antibody at 4 °C, followed by thrice washing in TBST buffer and further incubation in secondary antibody at room temperature for another 1 h. Subsequently, the membranes were washed again with TBST buffer and then developed with an enhanced chemiluminescence kit (PA112, Tiangen, China) using an ImageQuant LAS 4000 mini (GE Healthcare, USA). The primary antibodies used included anti-phospho-CD3ξ (Tyr142) (1:1000, PA5-37512, Thermo Fisher, USA), anti-total-CD3ξ (1:1000, 88083, Cell Signaling Technology, USA), anti-phospho-ZAP70 (Tyr493) (1:1000, AF3313, Affinity, USA), anti-total-ZAP70 (1:1000, 2705, Cell Signaling Technology, USA), anti-phospho-LCP2 (Ser376) (1:1000, 14745, Cell Signaling Technology, USA), anti-total-LCP2 (1:1000, 70896, Cell Signaling Technology, USA), and anti-GAPDH (1:2000, T0004, Affinity, USA). Images were analyzed using Image J.

**ELISA**

Transferrin in LM (plasma) and cytokines in the samples (cell supernatant of the in-vitro-treated human T cells) were measured using a human transferrin ELISA kit (SEC036Hu-96T, USCN, China), human IL-2 ELISA kit (SEA073Hu-96T, USCN, China), and human IFN-γ ELISA kit (SEA049Hu-96T, USCN, China) according to the manufacturers’ instructions, respectively. Transferrin in mice of LM (plasma) was measured using a mouse transferrin ELISA kit (CEC036Mu-96T, USCN, China) according to the manufacturers’ instructions.

**Statistical analysis**

The data obtained from independent experiments are presented as means ± standard deviation (SD). Results were analyzed using unpaired *t*-test or one-way analysis of variance (ANOVA) with Fisher’s protected *t*-tests in Prism 6 (GraphPad Software) and SPSS v22.0 (SPSS Inc, USA). Differences were considered significant at *p* < 0.05.

**Supplementary Figures**

**
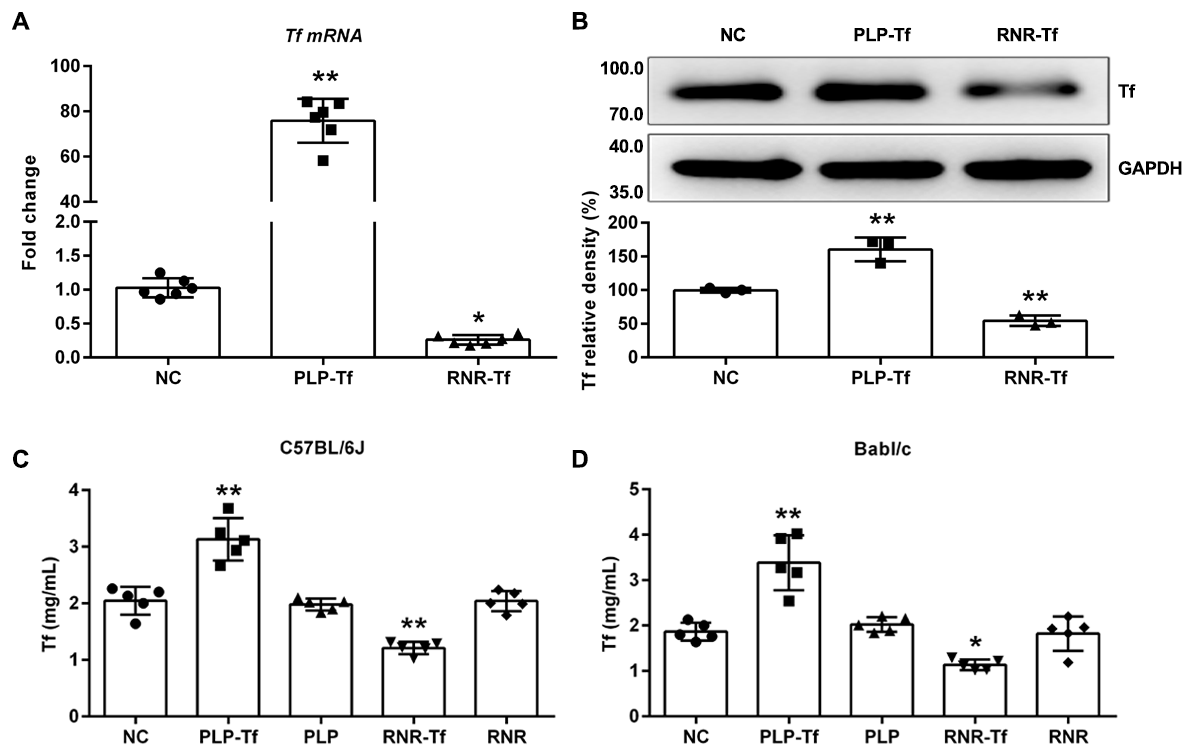
**

**Fig. S1 Construction of transferrin overexpression or knockdown vectors, related to Figure 2**

(A) *Transferrin* mRNA levels of BNL CL.2 cells after transfection of overexpression or knockdown plasmid of transferrin determined by qRT-PCR. Data represent means ± SD of six independent experiments, **p* < 0.05, ***p* < 0.01 by unpaired *t*-test.

(B) Transferrin levels in BNL CL.2 cells determined by Western blotting (top, Lane 1: control (Saline), Lane 2: overexpression (PLP-Tf), Lane 3: knockdown (RNR-Tf)). Quantification of Western blots is also shown (bottom). Data represent means ± SD of three independent experiments, ***p* < 0.01 by unpaired *t*-test.

(C-D) Plasma concentrations of transferrin in three groups of C57BL/6J mice (C) and BABL/c mice (D) (PLP-Tf and its blank PLP, RNR-Tf and its blank RNR, and control mice). Data represent means ± SD (*n* = 5), ***p* < 0.01 by unpaired *t*-test.

Tf: transferrin.

**
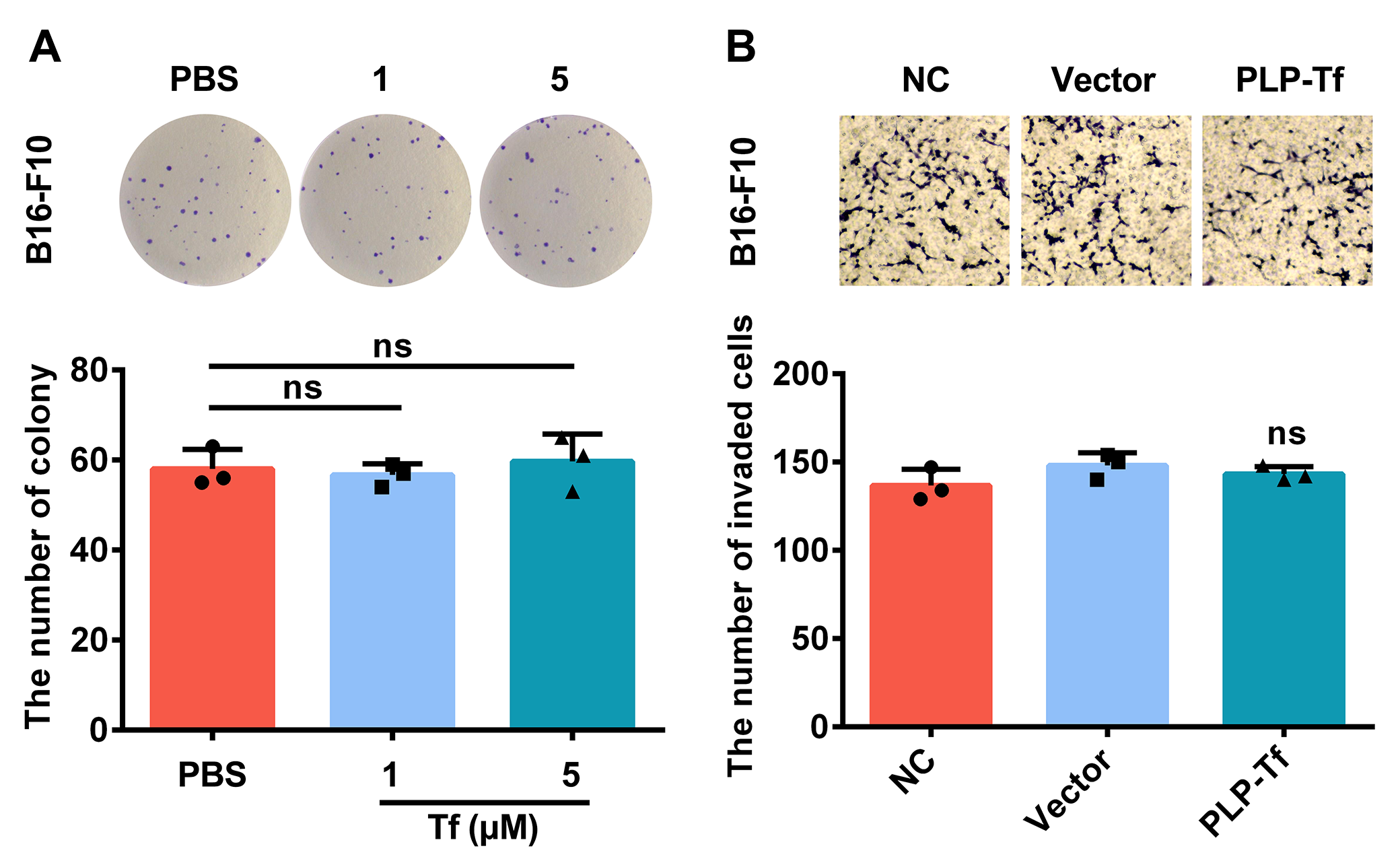
**

**Fig. S2 Transferrin have no effect on tumor cell colony formation and migration in *vitro***

(A) Cell colony formation analysis of the effect of Tf on B16F10 cells, Data represent mean ± SD of three independent experiments, ns, not significant by one-way ANOVA with Fisher’s protected *t* test.

(B) B16F10 cells were treated with PLP-Tf or vector for 2 days and then plated in transwell (8 µm) for migration assay. Data represent mean ± SD of three independent experiments, ns, not significant by one-way ANOVA with Fisher’s protected *t* test.

Tf: transferrin; PLP-Tf: Tf overexpression.


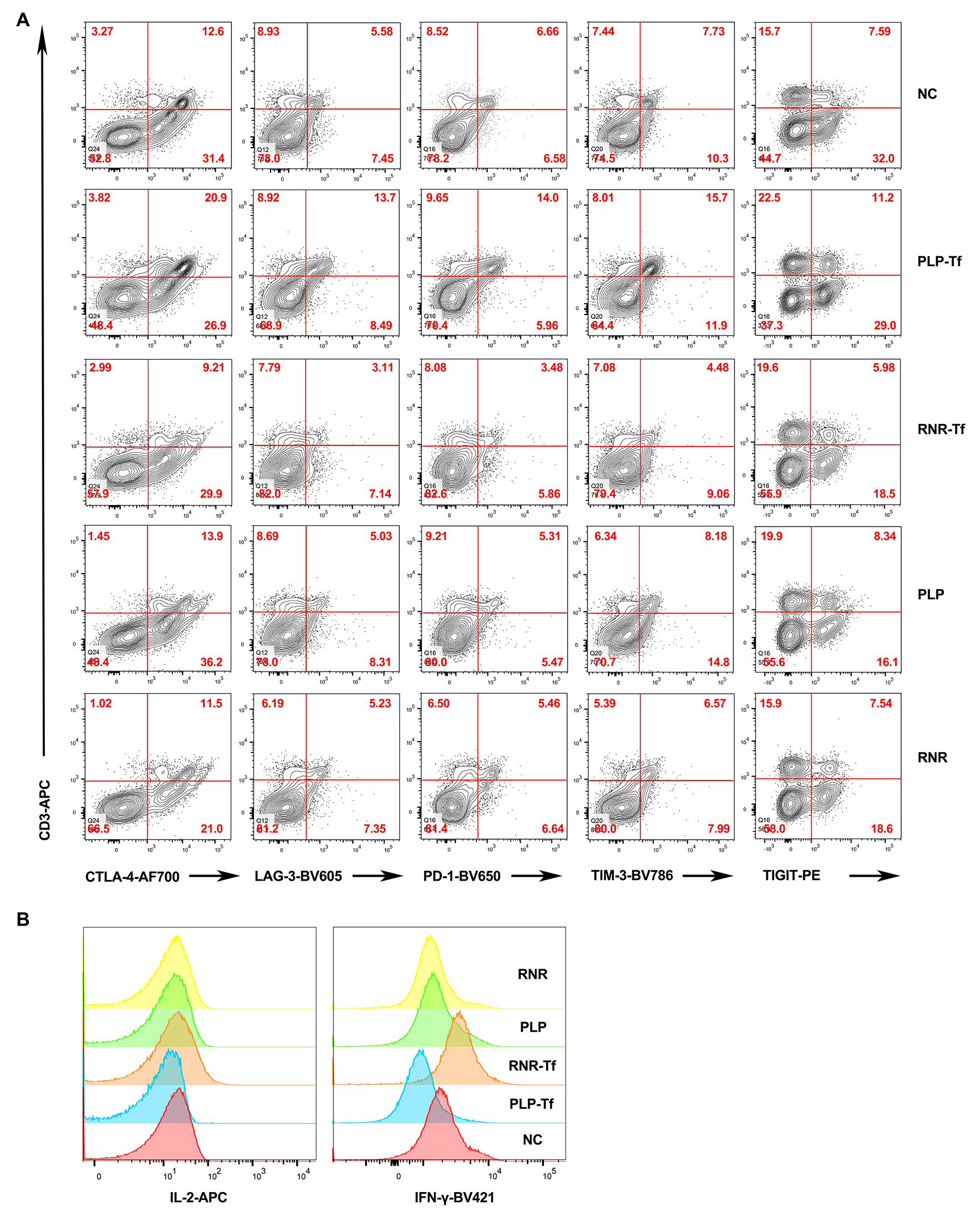


**Fig. S3 Transferrin overexpression and knockdown aggravates or attenuates T cell exhaustion of melanoma, respectively, related to Fig. 2**

(A) Flowcytometry analysis of the expression of CTLA-4, LAG-3, PD-1, TIM-3, and TIGIT in liver metastasis mice of B16F10 tumor.

(B) Flowcytometry analysis of the expression of IL-2 and IFN-γ in liver metastasis mice of B16F10 tumor.

Tf: transferrin; PLP-Tf: Tf overexpression and its blank PLP, RNR-Tf: Tf knockdown and its blank RNR.


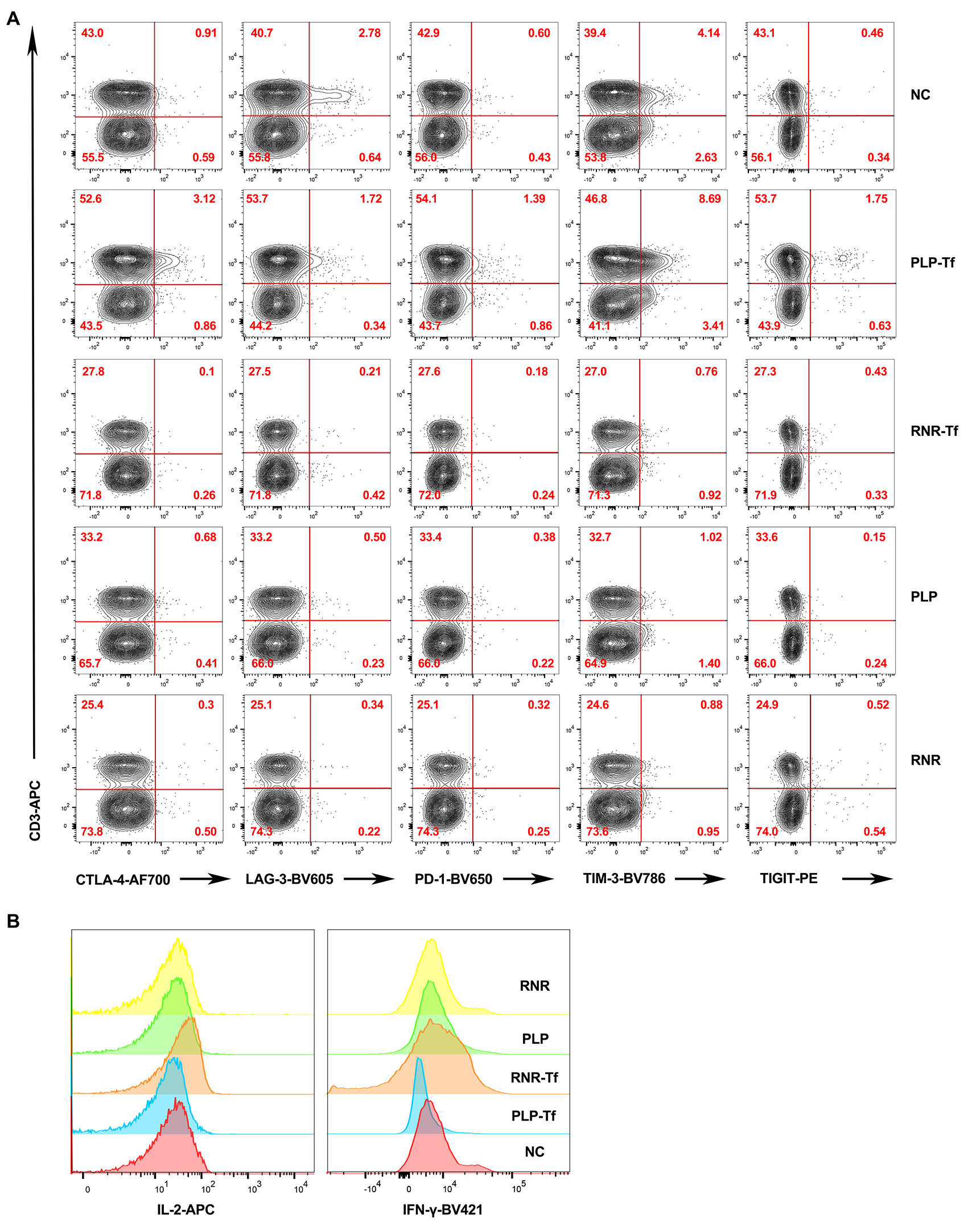


**Fig. S4 Transferrin overexpression and knockdown aggravates or attenuates T cell exhaustion of lymphoma, respectively, related to Fig. 2**

(A) Flowcytometry analysis of the expression of CTLA-4, LAG-3, PD-1, TIM-3, and TIGIT in liver metastasis mice of EL4 tumor.

(B) Flowcytometry analysis of the expression of IL-2 and IFN-γ in liver metastasis mice of EL4 tumor.

Tf: transferrin; PLP-Tf: Tf overexpression and its blank PLP, RNR-Tf: Tf knockdown and its blank RNR.


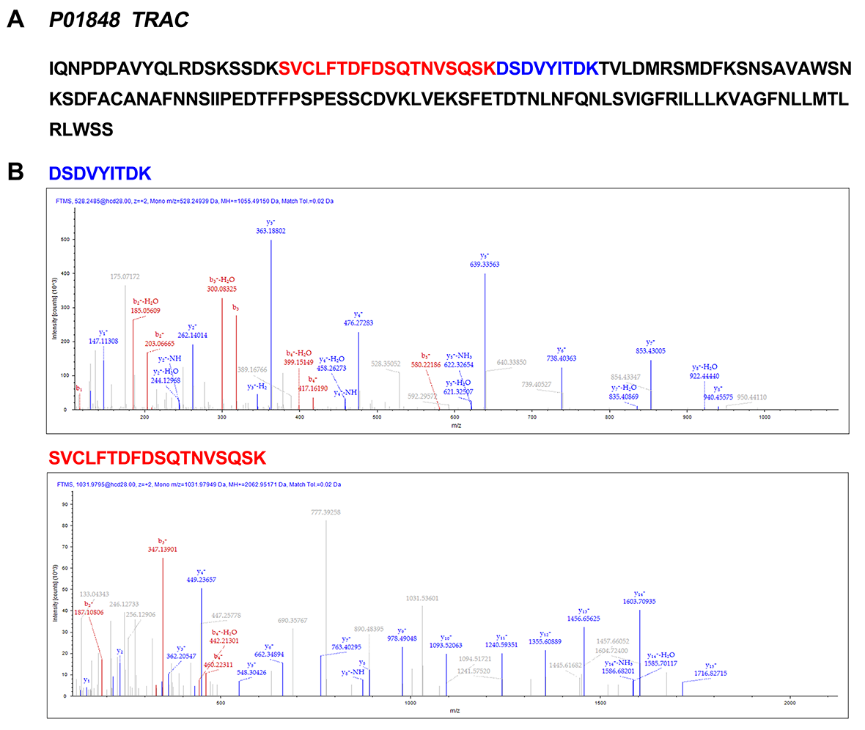


**Fig. S5 Identification of Tf-binding protein, αβTCR, related to Fig. 3**

(A) CoIP analysis of the interaction of protein with Tf in human PBMCs. LC-MS/MS analysis identified one Tf-binding protein, αβTCR.

(B) The secondary mass spectrometry analysis shows the peptides marked with blue and red in ‘A’.

Tf: transferrin; TRAC: T cell receptor α chain constant.

**
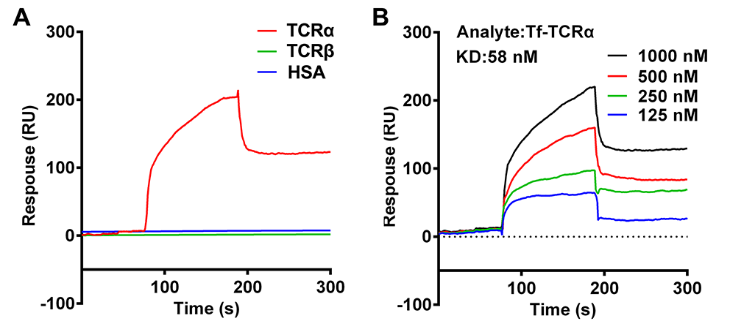
**

**Fig. S6 Transferrin binds to TCRα instead of TCRβ, related to Fig. 3**

(A) SPR analysis of the interaction of Tf with TCRα (1000 nM) and TCRβ (1000 nM), HSA (1000 nM) was the control. One representative experiment of three is shown.

(B) SPR analysis of the interaction of Tf with TCRα. One representative experiment of three is shown.

Tf: transferrin; HSA: human serum albumin.

**
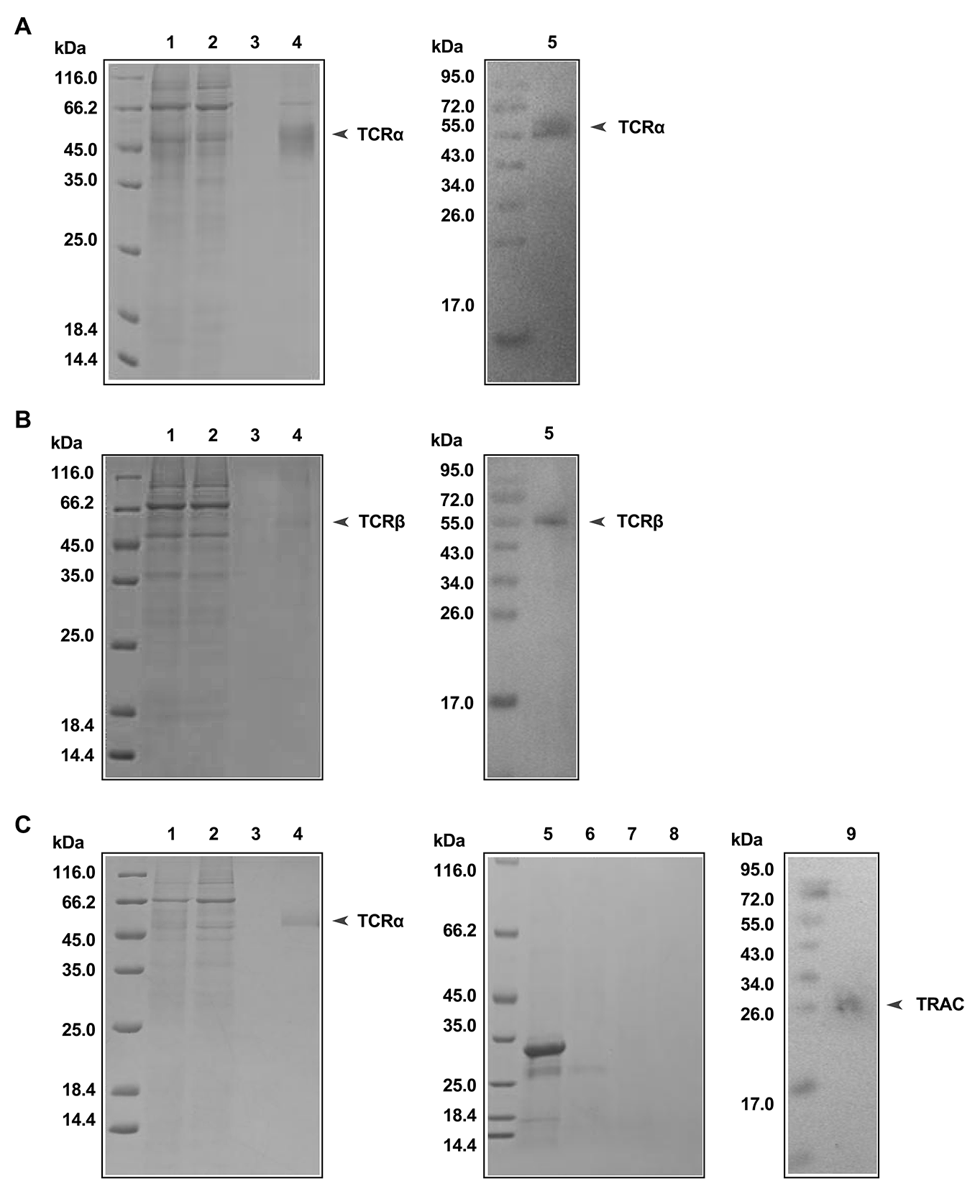
**

**Fig. S7 Expression and purification of TCRα, TCRβ and TRAC, related to Fig. 3**

(A-B) Expression and purification of TCRα and TCRβ. After transfection in HEK293F cells for 5 days, TCRα or TCRβ in the cell supernatant (Lanes 1) and fractions in flowing through (Lanes 2), washing (Lane 3: 20 mM imidazole buffer), and elution from Ni^2+^ affinity chromatography column by 500 mM (Lanes 4) imidazole elution buffer were analyzed by 12% SDS-PAGE with Coomassie-Blue staining (Left) and immunoblotting (right, Lanes 5: fractions eluted by 500 mM imidazole elution buffer).

(C) Expression and purification of TRAC. After transfecting in HEK293F cells for 5 days, Fc-TEV-TRAC in the cell supernatant (Lanes 1) and fraction in the flow-through (Lanes 2), washing (Lane 3: PBS buffer, pH 7.4), and elution with 0.1M glycine (pH 3.0) elution buffer from MabSelect SuRe LX column (Lanes 4) were analyzed by 12% SDS-PAGE with Coomassie-Blue staining (Left), followed by TEV protease digestion and purification using MabSelect SuRe LX column (Lane 5-8: middle), and immunoblotting (right, Lanes 9: flow-through fraction).

**
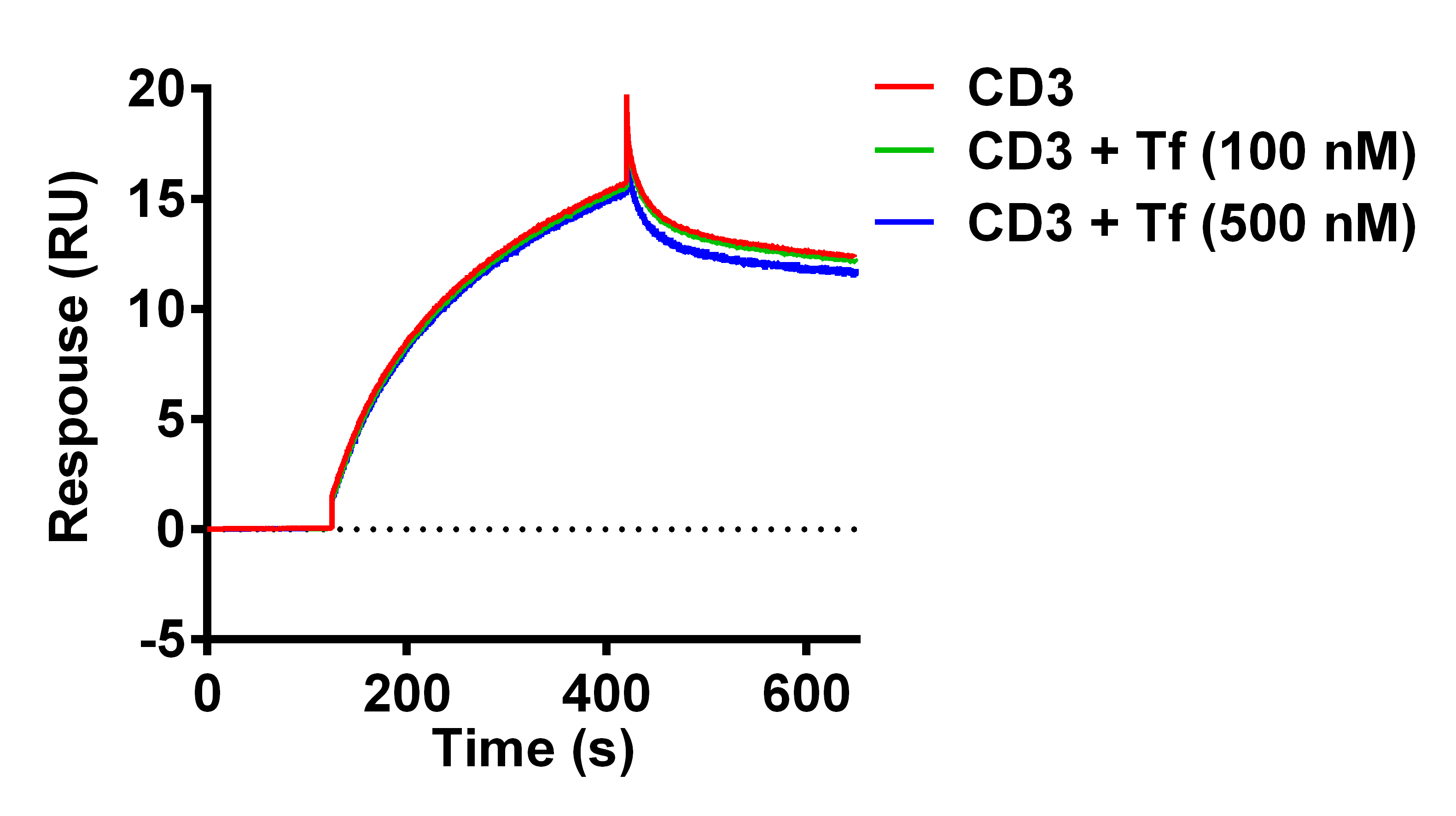
**

**Fig. S8 Transferrin did not inference anti-CD3 bind to CD3ԑ, related to Fig. 4**

**
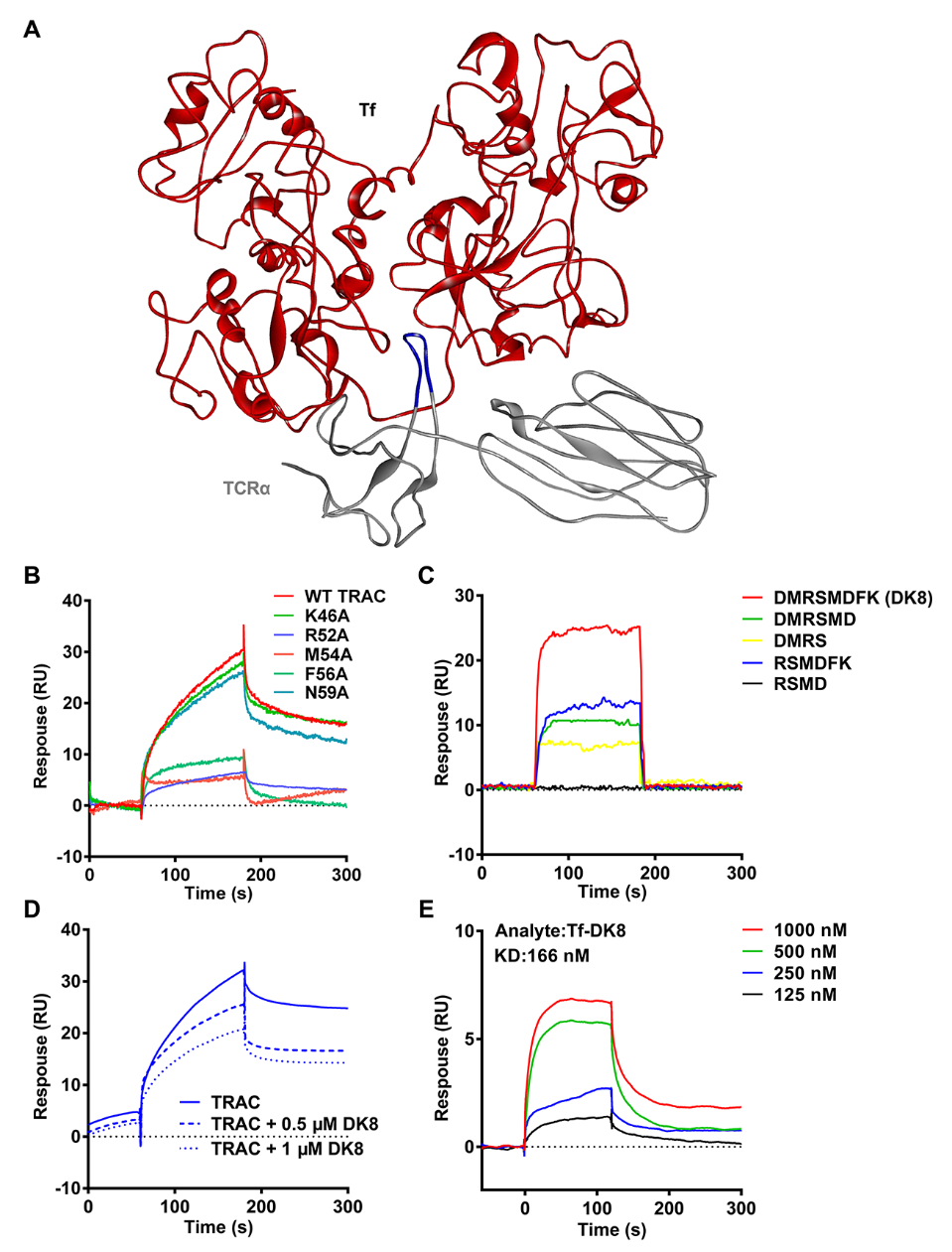
**

**Fig. S9 Analysis of key interaction sites between transferrin and TCRα, related to Fig. 5**

(A) 3D structure represents the interaction between Tf and TCRα by docking and molecular dynamics.

(B) SPR analysis of TRAC and its mutant sequences to Tf.

(C) SPR analysis of DK8 and its random sequences to Tf.

(D) SPR analysis of the interference of DK8 on Tf interaction with TRAC.

(E) SPR analysis of *KD* of DK8 to Tf.

Tf: transferrin; TRAC: T cell receptor α chain constant.

**
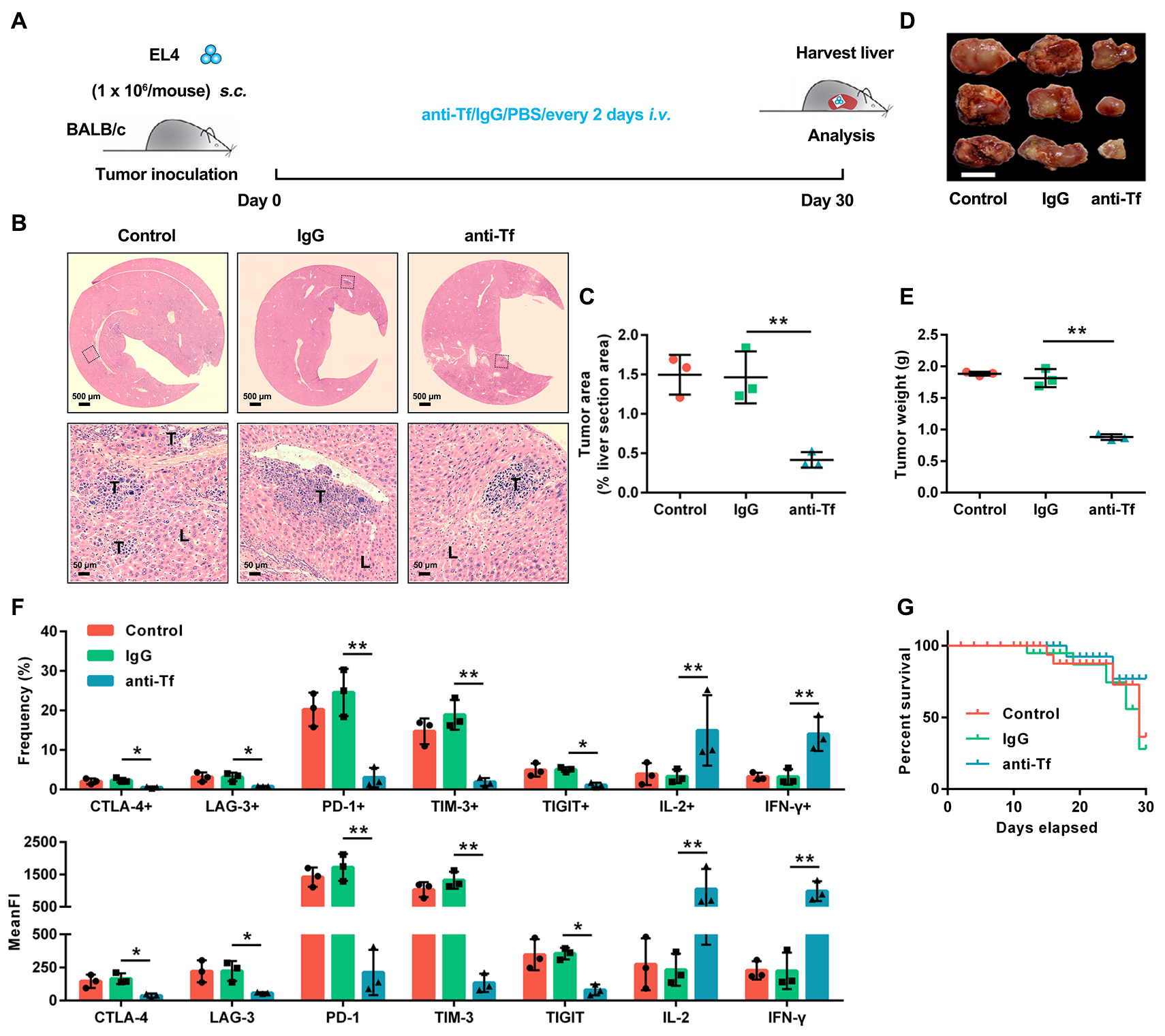
**

**Fig. S10 Therapeutic effects of Tf antibody on liver metastasis of EL4 tumor, related to Fig. 6**

(A) Schematic for BABL/c mouse model of Tf antibody intervention in liver metastasis of lymphoma. EL4 cells (1 × 10^6^) were first subcutaneously injected into BABL/c mice, followed by interventions (Tf antibody (50 μg per time *i.v.*), isotype IgG or PBS) every two days with an experimental cycle of 30 days.

(B-C) Hematoxylin-and-eosin (H&E) staining of liver sections (B) from liver metastasis mice of EL4 tumor, and tumor area was shown (C). T: tumor region, L: adjacent liver tissue; Scale bars, 500 or 50 μm. Images of one representative experiment of three are shown. Data represent mean ± SD of three independent experiments, ***p* < 0.01 by one-way ANOVA with Fisher’s protected *t* test.

(D-E) Representative images of tumors (D) and quantifications of tumor weights (E) in BABL/c mice (*n* = 3). Scale bar, 1 cm. Data represent mean ± SD of three independent experiments, ***p* < 0.01 by one-way ANOVA with Fisher’s protected *t* test.

(F) The frequency and MeanFI of CTLA-4, LAG-3, PD-1, TIM-3, TIGIT, IL-2 and IFN-γ T cells in CD3^+^ T cells in each population are summarized. Data represent means ± SD of three independent experiments. ns: no significance; **p* < 0.05, ***p* < 0.01 by one-way ANOVA with Fisher’s protected *t* test.

(G) Survival curve for tumor-bearing mice from indicated groups describe in (A) (*n* = 10/group).

Tf: transferrin.

**
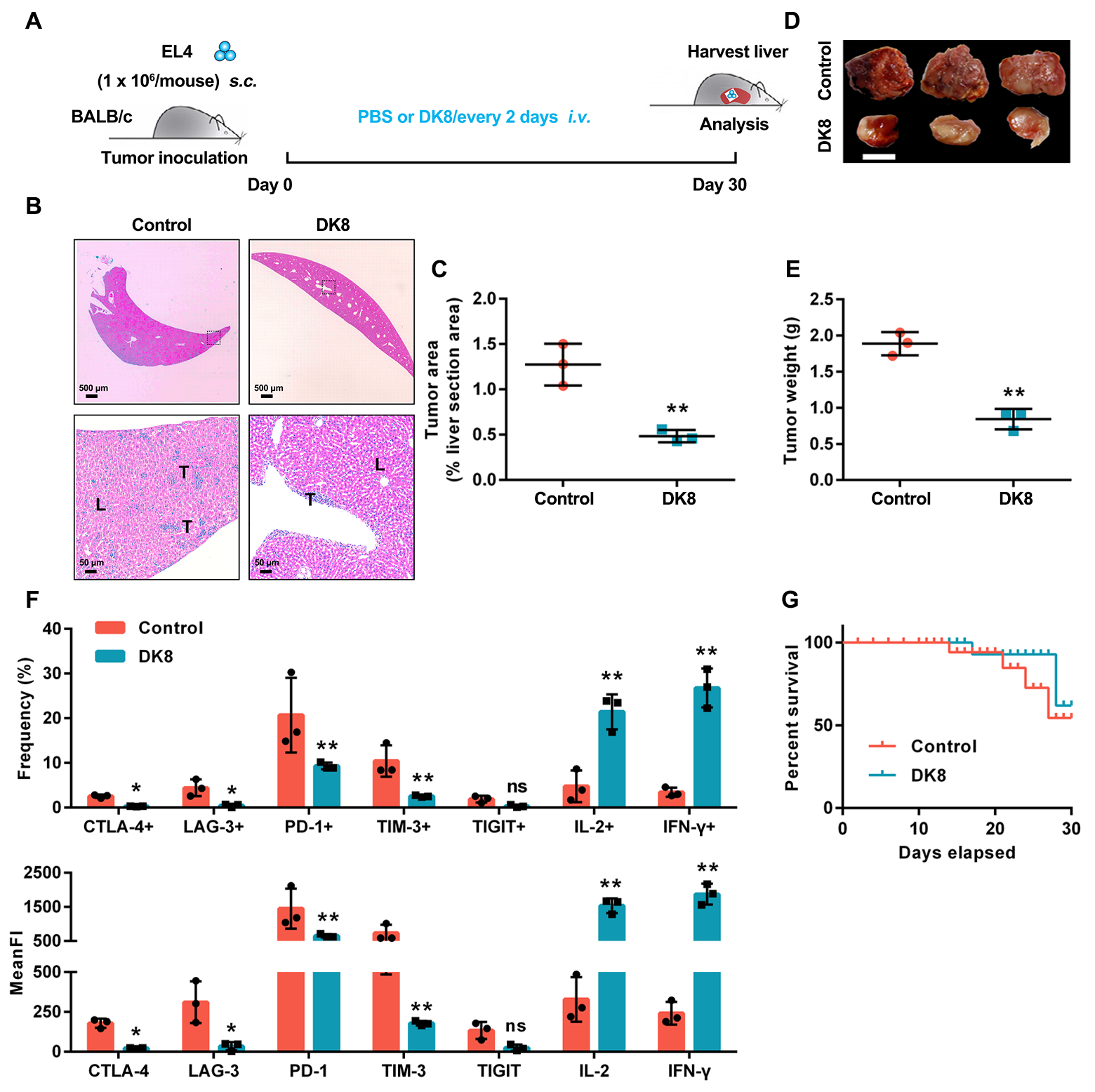
**

**Fig. S11 Therapeutic effects of interfering peptide DK8 on liver metastasis of EL4 tumor, related to Fig 6**

(A) Schematic for BABL/c mouse model of DK8 intervention in liver metastasis of lymphoma. EL4 cells (1 × 10^6^) were first subcutaneously injected into BABL/c mice, followed by interventions (DK8 (5 mg/kg *i.v.*) or PBS) every two days with an experimental cycle of 30 days.

(B-C) Hematoxylin-and-eosin (H&E) staining of liver sections (right) from liver metastasis mice of EL4 tumor, and tumor area was shown (left). T: tumor region, L: adjacent liver tissue; Scale bars, 500 or 50 μm. Images of one representative experiment of three are shown. Data represent mean ± SD of three independent experiments, ***p* < 0.01 by unpaired *t*-test.

(D-E) Representative images of tumors (D) and quantifications of tumor weights (E) in BABL/c mice (*n* = 3). Scale bar, 1 cm. Data represent mean ± SD of three independent experiments, ***p* < 0.01 by unpaired *t*-test.

(F) The frequency and MeanFI of CTLA-4, LAG-3, PD-1, TIM-3, TIGIT, IL-2 and IFN-γ T cells in CD3^+^ T cells are summarized in group of DK8 (blue) and control (red). Data represent means ± SD of three independent experiments. ns: no significance; **p* < 0.05, ***p* < 0.01 by one-way ANOVA with Fisher’s protected *t* test.

(G) Survival curve for tumor-bearing mice from indicated groups describe in (A) (*n* = 10/group).

Tf: transferrin.

**
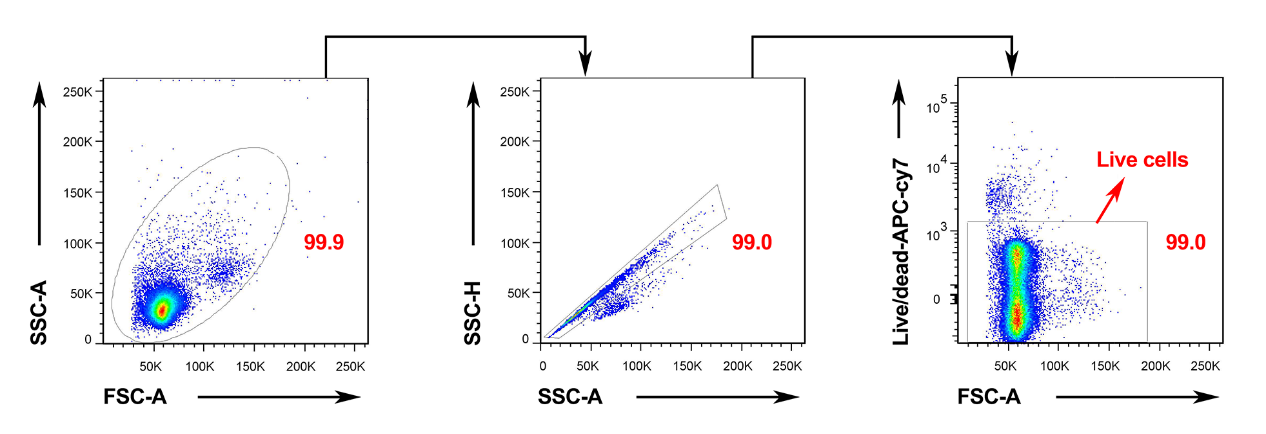
**

**Fig. S12 Gating strategy to analyze human or mice T cells**

**Supplementary Tables**

**Table S1 Case individual registration form**

| Group | Gender | Age (years) | TF (mg/mL) | Primary tumor site | Metastatic site |
| --- | --- | --- | --- | --- | --- |
| Patient | Female | 48 | 1.16 | Colon | Liver |
|  | Female | 48 | 3.78 | Rectum | Liver |
|  | Female | 47 | 2.66 | Rectum | Liver |
|  | Female | 52 | 2.73 | Uterine | Liver |
|  | Male | 48 | 3.74 | Rectum | Liver |
|  | Female | 50 | 1.50 | Rectum | Liver |
|  | Male | 58 | 1.72 | Stomach | Liver |
|  | Female | 36 | 1.18 | Breast | Liver |
|  | Female | 34 | 1.21 | Pancreas | Liver |
|  | Female | 39 | 1.16 | Breast | Liver |
|  | Male | 65 | 2.66 | Rectum | Liver |
|  | Female | 63 | 1.23 | Colon | Liver |
|  | Male | 46 | 1.50 | Colon | Liver |
|  | Male | 65 | 1.72 | Rectum | Liver |
|  | Female | 75 | 3.28 | Rectum | Liver |
|  | Female | 69 | 3.24 | Pancreas | Liver |
|  | Male | 62 | 1.17 | Colon | Liver |
|  | Female | 58 | 1.72 | Uterine | Liver |
|  | Male | 54 | 1.19 | Rectum | Liver |
|  | Female | 69 | 1.16 | Rectum | Liver |
|  | Female | 60 | 2.76 | Breast | Liver |
|  | Female | 52 | 2.66 | Rectum | Liver |
|  | Male | 51 | 2.68 | Colon | Liver |
|  | Male | 38 | 2.74 | Rectum | Liver |
|  | Male | 70 | 1.72 | Rectum | Liver |
|  | Female | 65 | 3.23 | Colon | Liver |
|  | Female | 57 | 1.05 | Liver | NA |
|  | Female | 59 | 1.84 | Liver | NA |
|  | Female | 42 | 1.05 | Liver | NA |
|  | Female | 70 | 1.01 | Liver | NA |
|  | Male | 52 | 1.28 | Liver | NA |
|  | Female | 59 | 0.64 | Liver | NA |
|  | Male | 65 | 0.87 | Liver | NA |
|  | Male | 52 | 1.46 | Liver | NA |
|  | Male | 86 | 1.06 | Liver | NA |
|  | Female | 40 | 0.87 | Liver | NA |
|  | Male | 59 | 1.19 | Liver | NA |
|  | Male | 54 | 1.04 | Liver | NA |
|  | Male | 58 | 1.32 | Liver | NA |
|  | Female | 37 | 1.22 | Liver | NA |
|  | Male | 29 | 1.08 | Liver | NA |
|  | Male | 43 | 1.07 | Liver | NA |
|  | Female | 47 | 1.68 | Liver | NA |
|  | Male | 67 | 1.05 | Liver | NA |
|  | Male | 80 | 1.11 | Liver | NA |
|  | Male | 31 | 1.18 | Liver | NA |
| Normal | Male | 66 | 1.72 | NA | NA |
|  | Female | 69 | 1.28 | NA | NA |
|  | Male | 52 | 1.31 | NA | NA |
|  | Female | 61 | 1.65 | NA | NA |
|  | Male | 39 | 1.05 | NA | NA |
|  | Male | 55 | 1.35 | NA | NA |
|  | Male | 49 | 1.35 | NA | NA |
|  | Male | 56 | 1.65 | NA | NA |
|  | Male | 60 | 1.11 | NA | NA |
|  | Male | 60 | 1.31 | NA | NA |
|  | Male | 44 | 0.51 | NA | NA |
|  | Male | 51 | 1.05 | NA | NA |
|  | Male | 53 | 1.21 | NA | NA |
|  | Female | 72 | 1.46 | NA | NA |
|  | Male | 55 | 1.88 | NA | NA |
|  | Female | 77 | 1.75 | NA | NA |
|  | Female | 55 | 1.66 | NA | NA |
|  | Male | 60 | 1.65 | NA | NA |
|  | Male | 61 | 1.65 | NA | NA |
|  | Female | 59 | 1.28 | NA | NA |
|  | Female | 55 | 1.36 | NA | NA |
|  | Male | 50 | 1.05 | NA | NA |
|  | Male | 53 | 1.30 | NA | NA |
|  | Male | 51 | 1.35 | NA | NA |

TF, transferrin; NA, not applicable.

**Table S2 Liver metastases patients in the cBioportal database**

| Sample ID | Age  (years) | Metastatic site | Primary tumor  site | Overall survival  (months) | Overall survival  status | TF |
| --- | --- | --- | --- | --- | --- | --- |
| 11004 | 30 | Liver | Colon | 8.11 | 1:DECEASED | 6.0636 |
| 11307 | 48 | Liver | Breast | 27.46 | 1:DECEASED | 147.1718 |
| 13261 | 44 | Liver | Sinus | 53.68 | 1:DECEASED | 36.8902 |
| 13577 | 51 | Liver | Lung | 27.69 | 1:DECEASED | 47.3352 |
| 13904 | 58 | Liver | Colon | 18.5 | 1:DECEASED | 463.4237 |
| 13931 | 33 | Liver | Bile Duct | 9.13 | 1:DECEASED | 116.1205 |
| 13947 | 38 | Liver | Appendix | 36.63 | 1:DECEASED | 0.3738 |
| 14232 | 63 | Liver | Colon | 23.36 | 1:DECEASED | 0.9468 |
| 15227 | 50 | Liver | Breast | 24.15 | 1:DECEASED | 12.6704 |
| 16385 | 44 | Liver | Pancreas | 11.66 | 1:DECEASED | 36.511 |
| 16500 | 68 | Liver | Lung | 23.65 | 1:DECEASED | 0.2536 |
| 16949 | 51 | Liver | Prostate | 16.62 | 1:DECEASED | 48.4341 |
| 17584 | 36 | Liver | Breast | 30.85 | 1:DECEASED | 19.9684 |
| 18154 | 39 | Liver | Breast | 43.73 | 1:DECEASED | 1.2574 |
| 18625 | 58 | Liver | Breast | 37.09 | 1:DECEASED | 1.5211 |
| 18880 | 60 | Liver | Breast | 48.95 | 1:DECEASED | 2.3445 |
| 19512 | 46 | Liver | Breast | 77.46 | 1:DECEASED | 47.5714 |
| 21347 | 50 | Liver | Breast | 54.5 | 1:DECEASED | 54.4619 |
| 22597 | 75 | Liver | Breast | 54.34 | 0:LIVING | 0.4191 |
| 23237 | 42 | Liver | Lung | 38.47 | 1:DECEASED | 3.2611 |
| 23239 | 66 | Liver | Eye | 78.84 | 0:LIVING | 0.1551 |
| 23315 | 55 | Liver | Colon | 35.15 | 1:DECEASED | 0.3203 |
| 23411 | 66 | Liver | NA | 33.25 | 1:DECEASED | 0.0873 |
| 23453 | 61 | Liver | Colon | 41.72 | 1:DECEASED | 2.4957 |
| 23454 | 62 | Liver | Breast | 30.06 | 1:DECEASED | 5.3355 |
| 23701 | 31 | Liver | Breast | 23.09 | 1:DECEASED | 0.4029 |
| 23706 | 65 | Liver | Breast | 60.22 | 1:DECEASED | 34.8469 |
| 23739 | 61 | Liver | Anus | 24.51 | 1:DECEASED | 27.6382 |
| 23764 | 60 | Liver | NA | 43.23 | 1:DECEASED | 78.2393 |
| 24045 | 75 | Liver | Breast | 27.3 | 1:DECEASED | 71.548 |
| 24077 | 59 | Liver | Colon | 37.06 | 1:DECEASED | 88.3537 |
| 24229 | 46 | Liver | Pancreas | 7.75 | 1:DECEASED | 0.3032 |
| 24243 | 34 | Liver | Pancreas | 6.93 | 1:DECEASED | 0 |
| 24358 | 48 | Liver | Gastroesophageal Junction | 5.58 | 1:DECEASED | 156.6925 |
| 24363 | 47 | Liver | Pancreas | 16.82 | 1:DECEASED | 20.1126 |
| 24423 | 51 | Liver | Lung | 22.77 | 1:DECEASED | 6.4293 |
| 24550 | 61 | Liver | Breast | 2.17 | 1:DECEASED | 3.9227 |
| 24838 | 70 | Liver | Breast | 32.23 | 1:DECEASED | 0.2529 |
| 25318 | 67 | Liver | Pancreas | 9.13 | 1:DECEASED | 4.768 |
| 25356 | 66 | Liver | Breast | 12.45 | 1:DECEASED | 48.1713 |
| 25419 | 49 | Liver | Colon | 10.74 | 1:DECEASED | 281.7648 |
| 25424 | 46 | Liver | Colon | 43.96 | 1:DECEASED | 71.6286 |
| 25435 | 59 | Liver | Lung | 10.74 | 1:DECEASED | 0.4036 |
| 25436 | 67 | Liver | Stomach | 18.89 | 1:DECEASED | 307.885 |
| 25437 | 77 | Liver | Colon | 44.38 | 0:LIVING | 18.6111 |
| 25440 | 54 | Liver | Colon | 46.81 | 1:DECEASED | 22.9389 |
| 25494 | 69 | Liver | Pancreas | 5.62 | 1:DECEASED | 78.7827 |
| 25496 | 52 | Liver | Pancreas | 61.01 | 0:LIVING | 14.7172 |
| 25497 | 59 | Liver | Colon | 49.7 | 1:DECEASED | 37.4273 |
| 25498 | 54 | Liver | Colon | 22.27 | 1:DECEASED | 6.3485 |
| 25500 | 51 | Liver | Stomach | 12.98 | 1:DECEASED | 770.2833 |
| 25535 | 39 | Liver | Bile Duct | 39.36 | 1:DECEASED | 11.6513 |
| 25582 | 62 | Liver | Pancreas | 6.77 | 1:DECEASED | 1.0487 |
| 25590 | 69 | Liver | Colon | 59.23 | 1:DECEASED | 0.8108 |
| 25593 | 32 | Liver | Lung | 30.35 | 1:DECEASED | 85.496 |
| 25594 | 65 | Liver | Stomach | 3.65 | 1:DECEASED | 77.9147 |
| 25604 | 40 | Liver | NA | 179.24 | 1:DECEASED | 68.8746 |
| 25662 | 40 | Liver | Breast | 3.06 | 1:DECEASED | 1.3183 |
| 25664 | 64 | Liver | Pancreas | 11.01 | 1:DECEASED | 13.1437 |
| 25719 | 63 | Liver | Colon | 16.2 | 1:DECEASED | 690.1609 |
| 25720 | 73 | Liver | Prostate | 46.19 | 0:LIVING | 0.1807 |
| 25731 | 63 | Liver | Bile Duct | 18.82 | 1:DECEASED | 1.4695 |
| 25751 | 63 | Liver | Bile Duct | 10.84 | 1:DECEASED | 83.4822 |
| 25753 | 58 | Liver | Colon | 30.22 | 1:DECEASED | 33.3445 |
| 25755 | 60 | Liver | Colon | 27.07 | 1:DECEASED | 7.1933 |
| 25767 | 50 | Liver | Pancreas | 13.73 | 1:DECEASED | 12.7547 |
| 25768 | 66 | Liver | Liver | 45.14 | 0:LIVING | 623.8866 |
| 25890 | 61 | Liver | Lung | 12.32 | 1:DECEASED | 2.2011 |
| 25899 | 67 | Liver | Pancreas | 7.92 | 1:DECEASED | 5.7617 |
| 25902 | 78 | Liver | Breast | 17.54 | 1:DECEASED | 170.2526 |
| 25960 | 72 | Liver | Colon | 55.55 | 1:DECEASED | 63.1678 |
| 25961 | 64 | Liver | Bile Duct | 15.41 | 1:DECEASED | 61.2021 |
| 25962 | 62 | Liver | Breast | 33.84 | 1:DECEASED | 0.6216 |
| 25965 | 49 | Liver | Anus | 11.43 | 1:DECEASED | 74.2592 |
| 25981 | 77 | Liver | Colon | 19.02 | 1:DECEASED | 8.5876 |
| 25982 | 58 | Liver | Breast | 7.26 | 1:DECEASED | 102.9639 |
| 25984 | 57 | Liver | Breast | 16.33 | 1:DECEASED | 760.0609 |
| 25989 | 55 | Liver | Ovary | 38.73 | 1:DECEASED | 0.2142 |
| 25990 | 58 | Liver | Breast | 41.85 | 0:LIVING | 1.0442 |
| 26017 | 38 | Liver | Thyroid | 100.76 | 1:DECEASED | 581.2643 |
| 26051 | 77 | Liver | Colon | 4.53 | 1:DECEASED | 235.9806 |
| 26124 | 65 | Liver | Colon | 46.32 | 1:DECEASED | 125.3484 |
| 26239 | 67 | Liver | Colon | 97.67 | 0:LIVING | 0.0457 |
| 26376 | 46 | Liver | Breast | 20.73 | 1:DECEASED | 52.3332 |
| 26409 | 67 | Liver | Lung | 20.66 | 1:DECEASED | 61.4706 |
| 26465 | 60 | Liver | Eye | 16.39 | 1:DECEASED | 33.5687 |
| 26850 | 45 | Liver | Breast | 41.16 | 0:LIVING | 26.5372 |
| 26851 | 66 | Liver | Colon | 76.05 | 1:DECEASED | 11.3849 |
| 27078 | 67 | Liver | Pancreas | 38.17 | 0:LIVING | 660.1349 |
| 27090 | 39 | Liver | Breast | 65.7 | 0:LIVING | 55.4741 |
| 27177 | 46 | Liver | Kidney | 8.44 | 1:DECEASED | 55.007 |
| 27178 | 57 | Liver | Gastroesophageal Junction | 5.49 | 1:DECEASED | 25.8926 |
| 27216 | 64 | Liver | Breast | 81.77 | 0:LIVING | 0.1001 |
| 27217 | 67 | Liver | Colon | 26.64 | 1:DECEASED | 76.8576 |
| 27329 | 54 | Liver | Breast | 34.53 | 1:DECEASED | 4.0089 |
| 27339 | 34 | Liver | Colon | 14.49 | 1:DECEASED | 130.7461 |
| 27519 | 52 | Liver | Pancreas | 14.59 | 1:DECEASED | 112.3556 |
| 27563 | 62 | Liver | Colon | 4.34 | 1:DECEASED | 50.414 |
| 27697 | 69 | Liver | Pancreas | 39.68 | 0:LIVING | 0.4276 |
| 28125 | 26 | Liver | Gastroesophageal Junction | 21.65 | 1:DECEASED | 0.2298 |
| 28130 | 37 | Liver | Pancreas | 8.34 | 1:DECEASED | 41.255 |
| 28212 | 51 | Liver | Pancreas | 6.21 | 1:DECEASED | 4.7116 |
| 28226 | 70 | Liver | Colon | 9.66 | 0:LIVING | 74.9834 |
| 28324 | 68 | Liver | Colon | 14.45 | 1:DECEASED | 3.4061 |
| 28325 | 57 | Liver | Breast | 62.16 | 1:DECEASED | 472.5819 |
| 28380 | 62 | Liver | Pancreas | 16.46 | 1:DECEASED | 532.6212 |
| 28382 | 60 | Liver | Breast | 52.63 | 1:DECEASED | 9.5739 |
| 28428 | 49 | Liver | Colon | 24.87 | 0:LIVING | 177.8865 |
| 28494 | 66 | Liver | Colon | 22.21 | 1:DECEASED | 27.4291 |
| 28496 | 45 | Liver | Colon | 16.23 | 0:LIVING | 0.2936 |
| 28665 | 51 | Liver | Breast | 38.9 | 1:DECEASED | 43.4607 |
| 28666 | 50 | Liver | Colon | 29.83 | 1:DECEASED | 98.0681 |
| 29146 | 59 | Liver | Colon | 10.68 | 1:DECEASED | 11.526 |
| 29147 | 70 | Liver | Lung | 190.14 | 1:DECEASED | 0.0452 |
| 29759 | 69 | Liver | Breast | 35.64 | 1:DECEASED | 138.2624 |
| 29766 | 52 | Liver | Appendix | 8.41 | 1:DECEASED | 0.3431 |
| 29999 | 40 | Liver | Colon | 28.91 | 1:DECEASED | 4.341 |
| 30000 | 59 | Liver | Lung | 28.94 | 0:LIVING | 0.8698 |
| 30082 | 62 | Liver | unknown | 11.17 | 1:DECEASED | 11.8303 |
| 30159 | 69 | Liver | Colon | 25.1 | 1:DECEASED | 25.016 |
| 30222 | 60 | Liver | Gastroesophageal Junction | 8.51 | 1:DECEASED | 148.9468 |
| 30251 | 53 | Liver | Colon | 16.66 | 1:DECEASED | 117.0958 |
| 30252 | 41 | Liver | Breast | 95.27 | 1:DECEASED | 0.3357 |
| 30321 | 64 | Liver | Colon | 27.37 | 1:DECEASED | 1.4419 |
| 30425 | 73 | Liver | Breast | 31.54 | 1:DECEASED | 239.2414 |
| 30431 | 24 | Liver | NA | 61.99 | 0:LIVING | 120.7684 |
| 30492 | 48 | Liver | Colon | 37.39 | 1:DECEASED | 87.5146 |
| 30497 | 76 | Liver | Colon | 16.33 | 1:DECEASED | 1.6126 |
| 30498 | 33 | Liver | Pancreas | 30.09 | 1:DECEASED | 11.3389 |
| 30504 | 41 | Liver | Colon | 14.13 | 1:DECEASED | 0.0987 |
| 30609 | 47 | Liver | Colon | 48.36 | 1:DECEASED | 1.6823 |
| 30635 | 62 | Liver | Colon | 45.4 | 0:LIVING | 184.5915 |
| 30784 | 70 | Liver | Ovary | 48.03 | 0:LIVING | 19.7192 |
| 30811 | 71 | Liver | Colon | 52.79 | 1:DECEASED | 79.4995 |
| 30902 | 36 | Liver | Breast | 29.57 | 1:DECEASED | 27.3666 |
| 31043 | 53 | Liver | Breast | 41.16 | 1:DECEASED | 195.6844 |
| 31184 | 48 | Liver | Skin | 19.68 | 1:DECEASED | 24.4183 |
| 31370 | 57 | Liver | Breast | 18.1 | 1:DECEASED | 94.0562 |
| 31509 | 65 | Liver | Breast | 27.43 | 1:DECEASED | 85.884 |
| 31870 | 61 | Liver | Small Bowel | 54.5 | 1:DECEASED | 629.2594 |
| 31923 | 45 | Liver | Breast | 27 | 0:LIVING | 50.4548 |
| 31982 | 55 | Liver | NA | 40.57 | 0:LIVING | 0.5644 |
| 32033 | 36 | Liver | Colon | 65.05 | 0:LIVING | 89.5679 |
| 32397 | 59 | Liver | Breast | 9.2 | 0:LIVING | 145.9437 |
| 32398 | 40 | Liver | Breast | 5.81 | 1:DECEASED | 148.4903 |
| 32408 | 69 | Liver | Pancreas | 15.05 | 1:DECEASED | 85.3952 |
| 32557 | 40 | Liver | Pancreas | 12.52 | 1:DECEASED | 85.0993 |
| 32571 | 32 | Liver | Breast | 27.99 | 0:LIVING | 659.3372 |
| 32573 | 51 | Liver | Colon | 28.06 | 0:LIVING | 186.1244 |
| 32656 | 48 | Liver | Pancreas | 16.79 | 1:DECEASED | 228.5 |
| 32667 | 58 | Liver | Eye | 10.94 | 1:DECEASED | 0.6392 |
| 32959 | 45 | Liver | Colon | 36.1 | 0:LIVING | 0.6133 |
| 32960 | 60 | Liver | Bile Duct | 2.6 | 1:DECEASED | 0.2646 |
| 32971 | 57 | Liver | Lung | 1.25 | 1:DECEASED | 16.34 |
| 32972 | 51 | Liver | Colon | 17.74 | 1:DECEASED | 498.7674 |
| 33129 | 54 | Liver | Pancreas | 5.35 | 1:DECEASED | 3.5246 |
| 33480 | 52 | Liver | Colon | 13.93 | 1:DECEASED | 65.2756 |
| 33740 | 72 | Liver | Stomach | 18 | 0:LIVING | 172.6389 |
| 34115 | 60 | Liver | Pancreas | 42.77 | 1:DECEASED | 134.2872 |
| 34120 | 54 | Liver | Breast | 35.97 | 1:DECEASED | 2.8892 |
| 34122 | 55 | Liver | Colon | 35.87 | 0:LIVING | 820.4577 |
| 34159 | 47 | Liver | Kidney | 27.2 | 0:LIVING | 0.4069 |
| 34288 | 67 | Liver | Pancreas | 8.84 | 1:DECEASED | 130.9309 |
| 34491 | 56 | Liver | Pancreas | 22.54 | 0:LIVING | 5.2465 |
| 35289 | 48 | Liver | Colon | 8.25 | 1:DECEASED | 168.0208 |
| 35290 | 86 | Liver | Eye | 10.74 | 1:DECEASED | 22.3555 |
| 35336 | 51 | Liver | Bile Duct | 15.87 | 1:DECEASED | 2.4551 |
| 36062 | 56 | Liver | Stomach | 13.27 | 1:DECEASED | 374.7081 |
| 36069 | 70 | Liver | Bile Duct | 5.39 | 1:DECEASED | 15.5343 |
| 36700 | 36 | Liver | Breast | 6.04 | 1:DECEASED | 6.8811 |
| 36701 | 42 | Liver | Pancreas | 20.63 | 0:LIVING | 73.7196 |
| 36748 | 56 | Liver | Pancreas | 21.85 | 0:LIVING | 135.8076 |
| 36749 | 67 | Liver | Eye | 43.63 | 0:LIVING | 3.2003 |
| 36750 | 58 | Liver | Esophagus | 8.61 | 1:DECEASED | 2.3817 |
| 37025 | 55 | Liver | Pancreas | 22.31 | 0:LIVING | 167.9462 |
| 37077 | 65 | Liver | Breast | 49.18 | 1:DECEASED | 23.8029 |
| 37080 | 70 | Liver | Colon | 28.12 | 1:DECEASED | 74.0058 |
| 37097 | 40 | Liver | Breast | 31.67 | 0:LIVING | 6.5171 |
| 37155 | 59 | Liver | Colon | 20.2 | 0:LIVING | 132.9953 |
| 37249 | 54 | Liver | Pancreas | 12.88 | 1:DECEASED | 52.4253 |
| 37297 | 55 | Liver | Gastroesophageal Junction | 11.3 | 1:DECEASED | 32.2592 |
| 37311 | 54 | Liver | Colon | 79.8 | 1:DECEASED | 246.7245 |
| 37365 | 54 | Liver | Breast | 17.31 | 1:DECEASED | 2.0239 |
| 37718 | 61 | Liver | Pancreas | 19.55 | 1:DECEASED | 0.1685 |
| 37720 | 71 | Liver | Colon | 31.31 | 1:DECEASED | 34.7412 |
| 37828 | 59 | Liver | Bile Duct | 10.78 | 1:DECEASED | 335.564 |
| 37844 | 70 | Liver | Breast | 121.58 | 1:DECEASED | 155.0278 |
| 37967 | 66 | Liver | Pancreas | 16.82 | 1:DECEASED | 227.5916 |
| 37970 | 59 | Liver | Bile Duct | 12.32 | 1:DECEASED | 65.7786 |
| 38228 | 42 | Liver | NA | 28.42 | 0:LIVING | 0.0681 |
| 38250 | 41 | Liver | Breast | 7.98 | 0:LIVING | 218.6832 |
| 38505 | 58 | Liver | Pancreas | 20.3 | 0:LIVING | 711.5108 |
| 38506 | 70 | Liver | Colon | 26.05 | 1:DECEASED | 1.231 |
| 38536 | 62 | Liver | Pancreas | 9.59 | 1:DECEASED | 127.2419 |
| 38537 | 49 | Liver | Pancreas | 12.55 | 1:DECEASED | 21.1193 |
| 38705 | 55 | Liver | Breast | 76.74 | 0:LIVING | 91.603 |
| 38706 | 72 | Liver | NA | 34.07 | 1:DECEASED | 0.302 |
| 38727 | 75 | Liver | Lung | 28.81 | 0:LIVING | 45.4755 |
| 38744 | 70 | Liver | Eye | 31.18 | 0:LIVING | 412.6528 |
| 38850 | 50 | Liver | Breast | 8.02 | 1:DECEASED | 30.8134 |
| 38887 | 53 | Liver | Breast | 48.32 | 0:LIVING | 4.1011 |

TF, transferrin; NA, not applicable.

**Supplementary References**

1. Tang X, Zhang Z, Fang M, Han Y, Wang G, Wang S, Xue M, Li Y, Zhang L, Wu J, et al: Transferrin plays a central role in coagulation balance by interacting with clotting factors. *Cell Res* 2020, 30**:**119-132.
