# Supplementary material for: Transferrin Disassociates TCR from CD3 Signaling Apparatus to Promote Metastasis
Source: Research (Wash D C). 2025 Jan 13;8:0578. doi: 10.34133/research.0578 (PMC11731779; doi:10.34133/research.0578)
Supplement: Supplementary 1 — Supplementary Methods Figs. S1 to S12 Tables S1 and S2 Supplementary Reference [file research.0578.f1.zip › Supplementary gels blot.pdf]

Full unedited gel for Fig. 1C

kDa

Fig. 1C-Tf

100.0

70.0

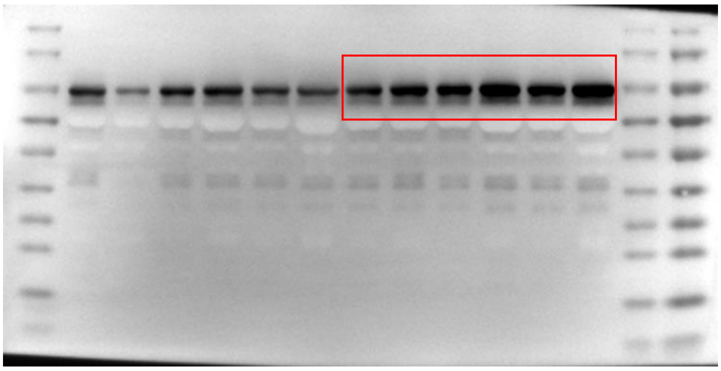

kDa

Fig. 1C-RP

100.0

70.0

55.0

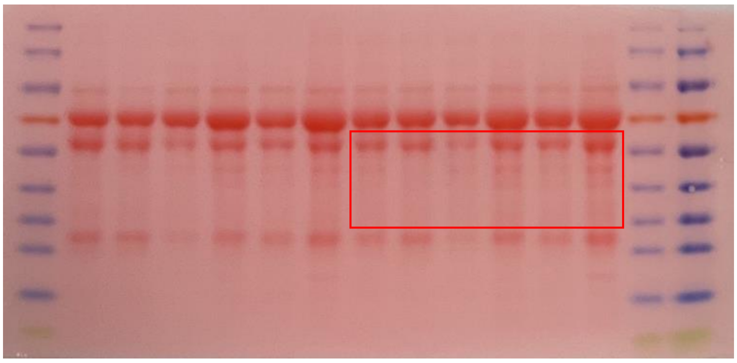

Full unedited gel for Fig. 1F

kDa

Fig. 1F-Tf

100.0
70.0

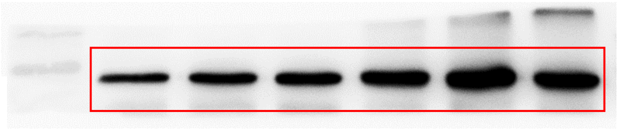

kDa

Fig. 1F-GAPDH

40.0

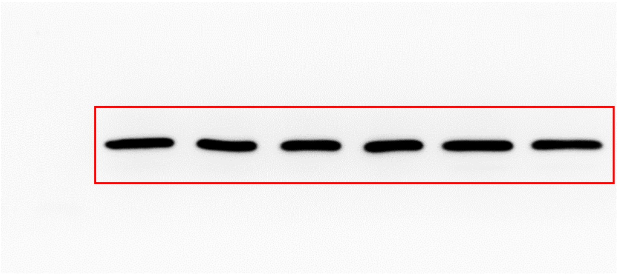

Full unedited gel for Fig. 3B

kDa Fig. 3B-Tf

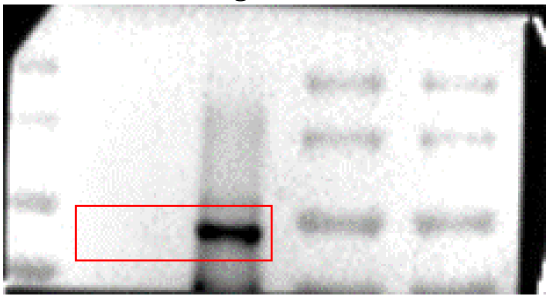

100.0
70.0

kDa Fig. 3B-TRAC

35.0
25.0

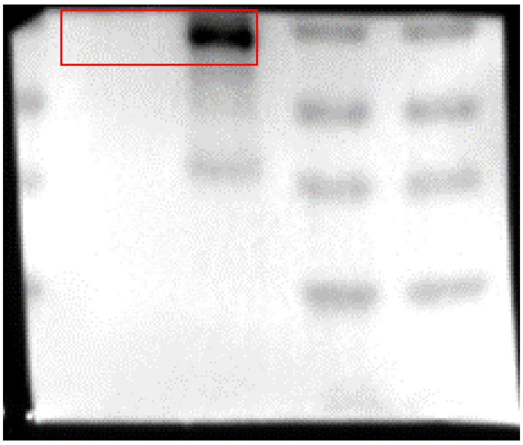

kDa Fig. 3B-input-TRAC

35.0

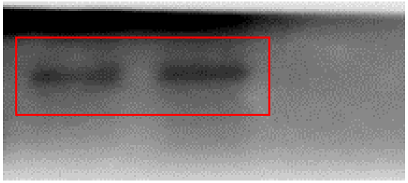

kDa Fig. 3B-input-GAPDH

40.0
35.0

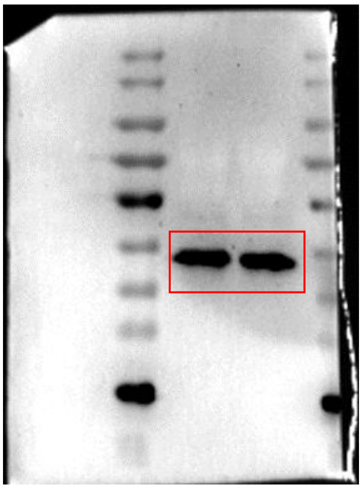

Full unedited gel for Fig. 3D

kDa Fig. 3D-CD3

25.0

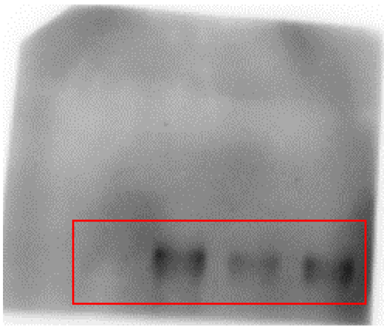

kDa Fig. 3D-TCRα

35.0

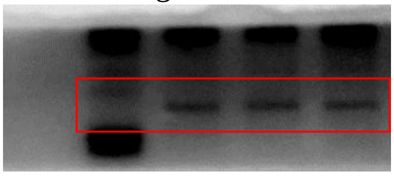

kDa Fig. 3D-input-TCRα

40.0
35.0

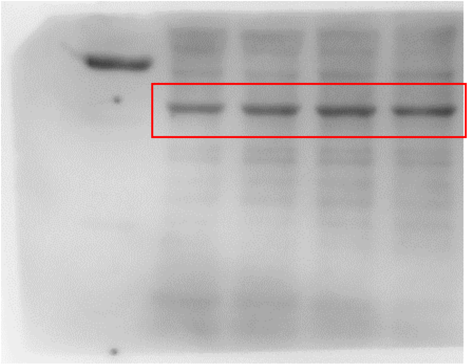

kDa Fig. 3D-input-GAPDH

40.0

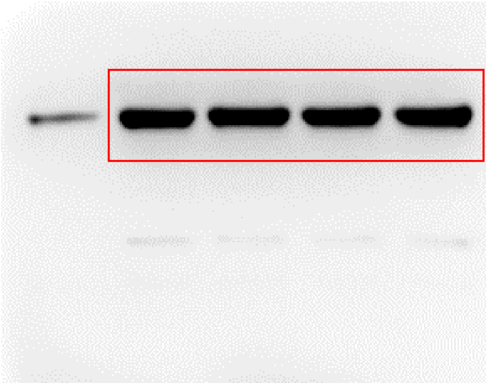

Full unedited gel for Fig. 4A

kDa Fig. 4A-p-CD3ξ

25.0

15.0

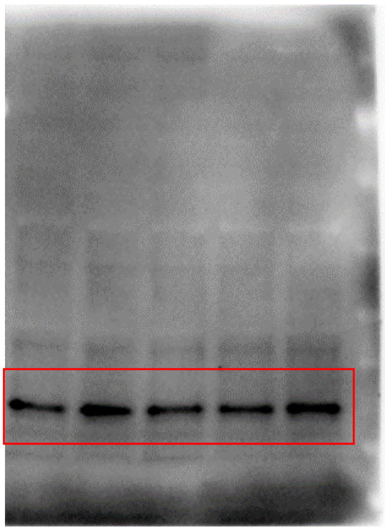

kDa Fig. 4A-CD3ξ

25.0

15.0

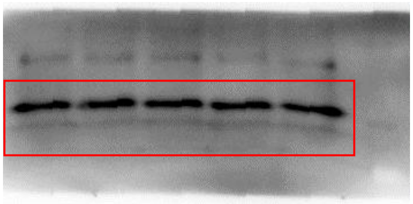

kDa Fig. 4A-p-ZAP70

70.0

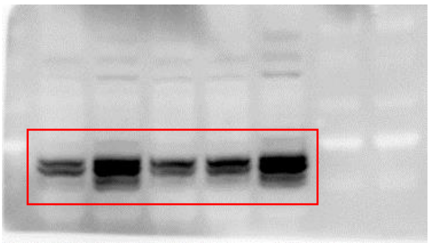

kDa

Fig. 4A-ZAP70

70.0

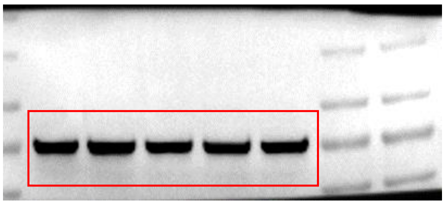

kDa

Fig. 4A-p-LCP2

100.0

70.0

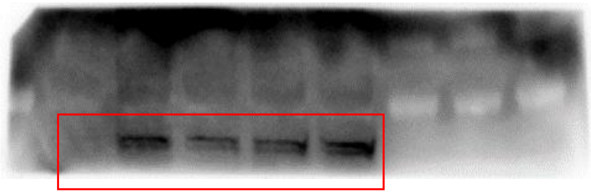

kDa

Fig. 4A-LCP2

100.0

70.0

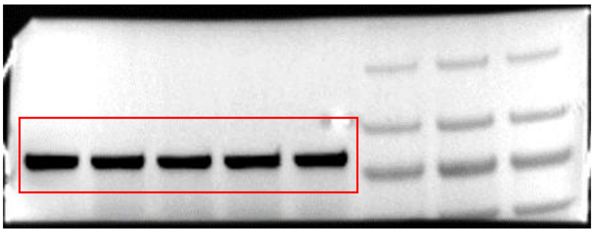

kDa

Fig. 4A-GAPDH

40.0

35.0

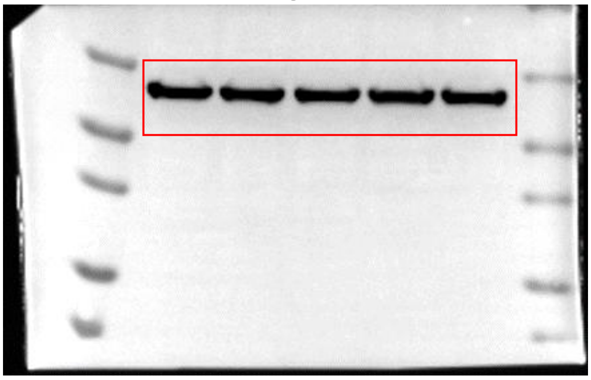

Full unedited gel for Fig. S1B

kDa Fig. S1B-Tf

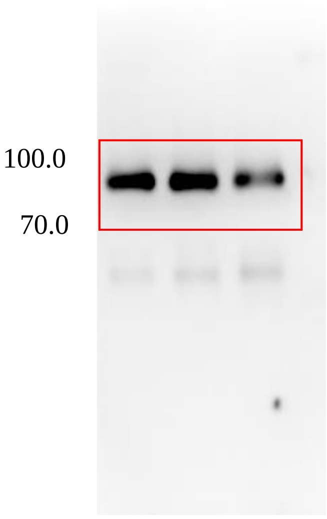

kDa Fig. S1B-GAPDH

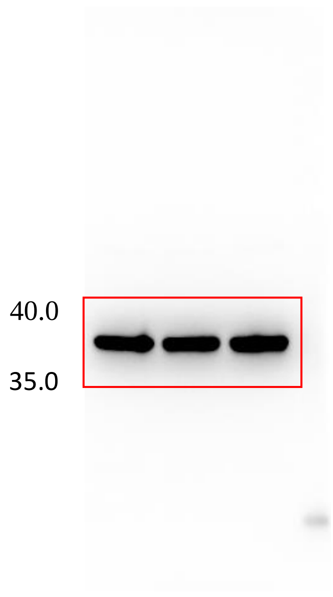

The red dashed square indicates where it was cropped
