# Supplementary figures and images for: Transferrin Disassociates TCR from CD3 Signaling Apparatus to Promote Metastasis
Source: Research (Wash D C). 2025 Jan 13;8:0578. doi: 10.34133/research.0578 (PMC11731779; doi:10.34133/research.0578)

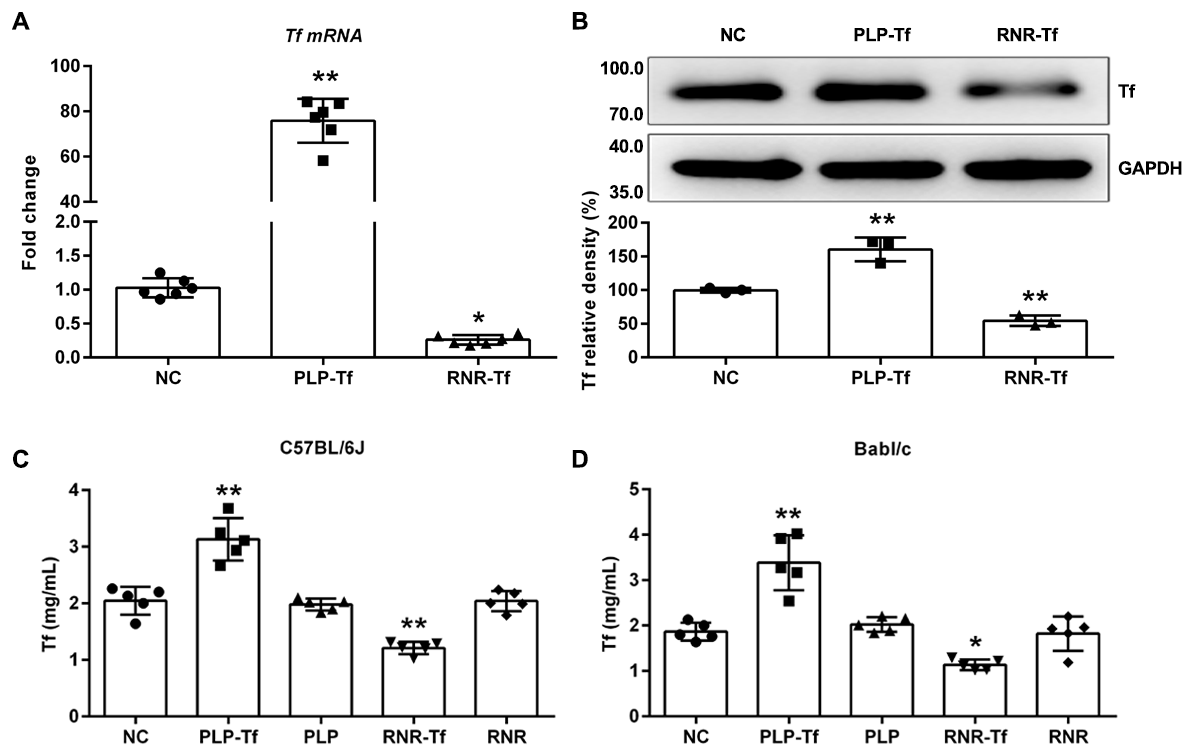

Supplement: Supplementary 1 — Supplementary Methods Figs. S1 to S12 Tables S1 and S2 Supplementary Reference [file research.0578.f1.zip › Fig S1.tif]

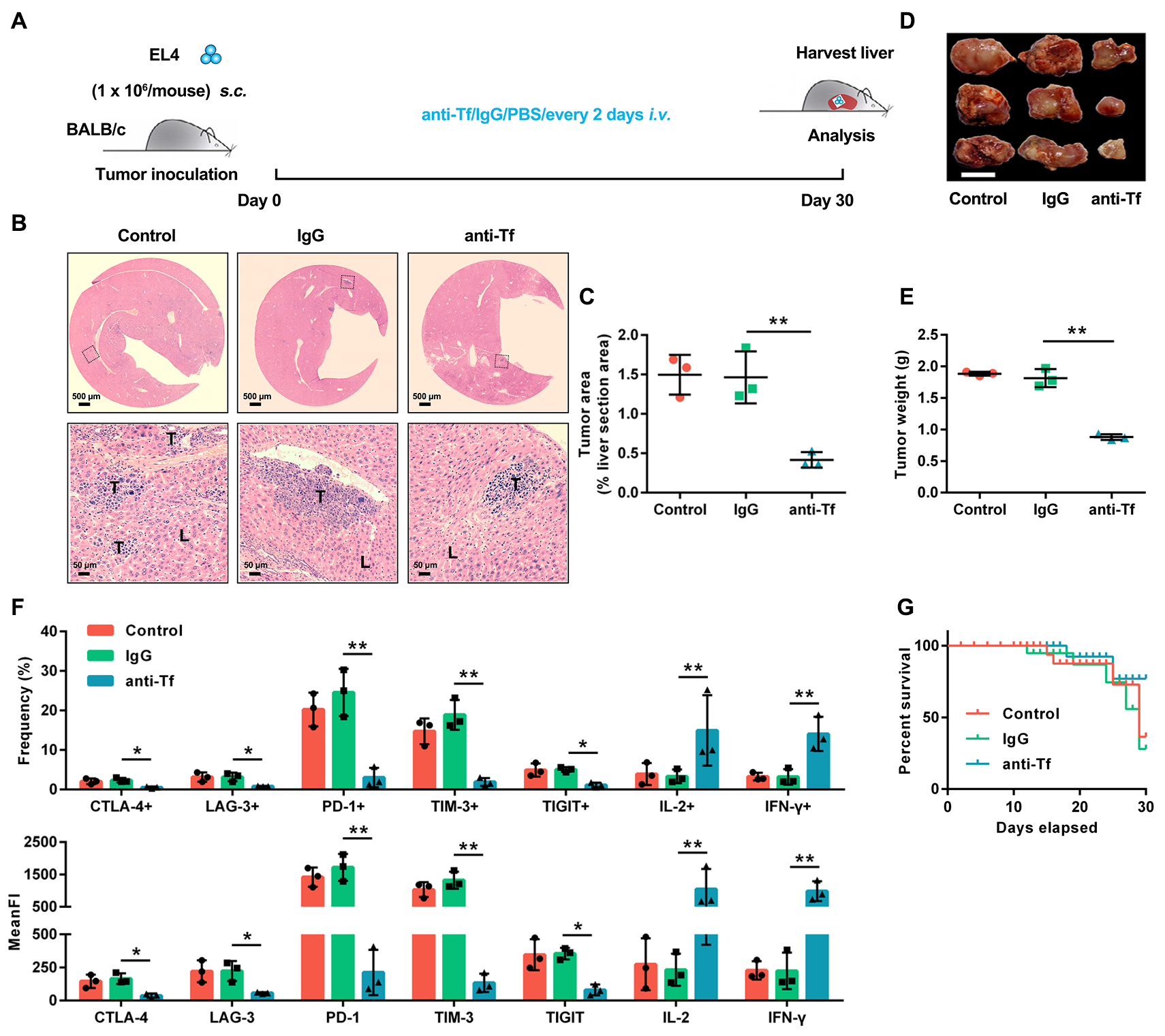

Supplement: Supplementary 1 — Supplementary Methods Figs. S1 to S12 Tables S1 and S2 Supplementary Reference [file research.0578.f1.zip › Fig S10.tif]

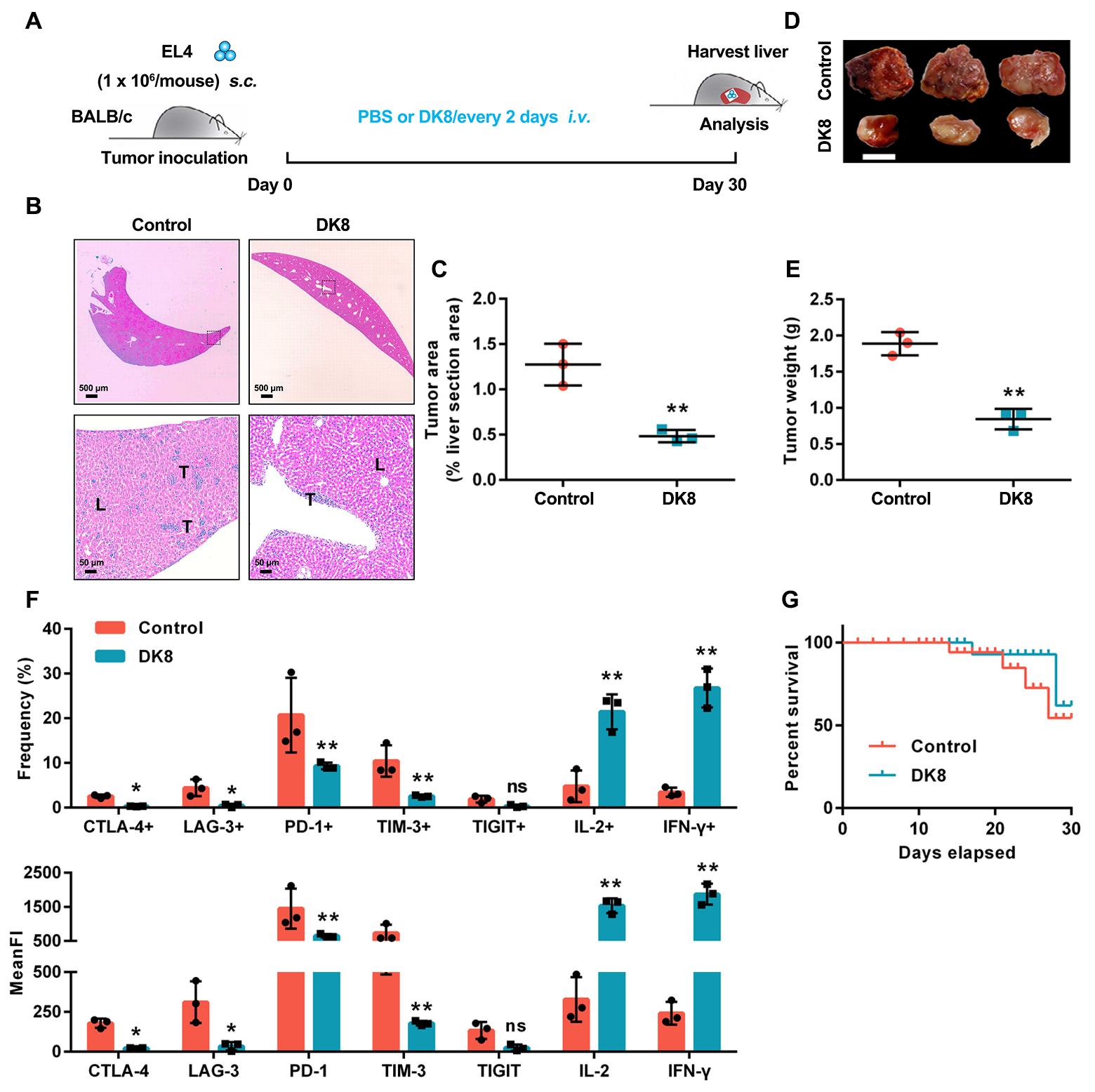

Supplement: Supplementary 1 — Supplementary Methods Figs. S1 to S12 Tables S1 and S2 Supplementary Reference [file research.0578.f1.zip › Fig S11.tif]

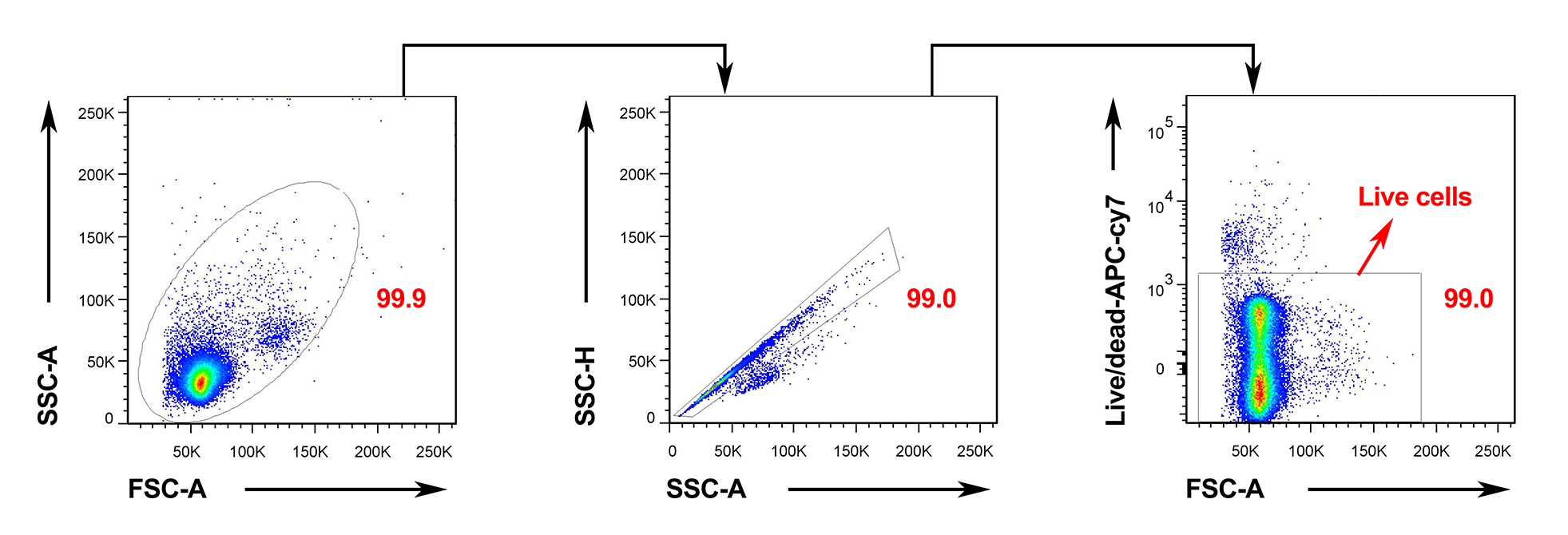

Supplement: Supplementary 1 — Supplementary Methods Figs. S1 to S12 Tables S1 and S2 Supplementary Reference [file research.0578.f1.zip › Fig S12.tif]

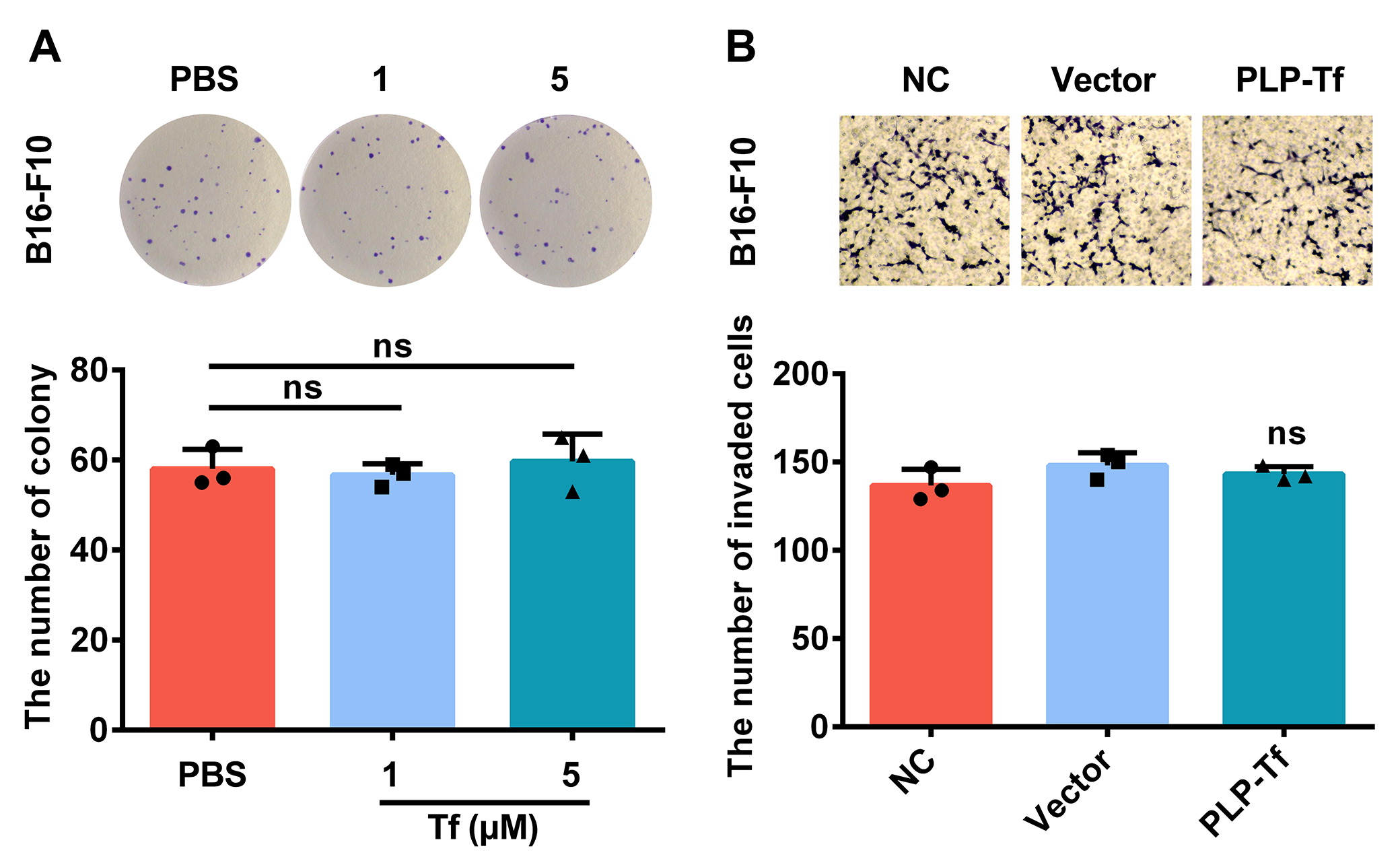

Supplement: Supplementary 1 — Supplementary Methods Figs. S1 to S12 Tables S1 and S2 Supplementary Reference [file research.0578.f1.zip › Fig S2.tif]

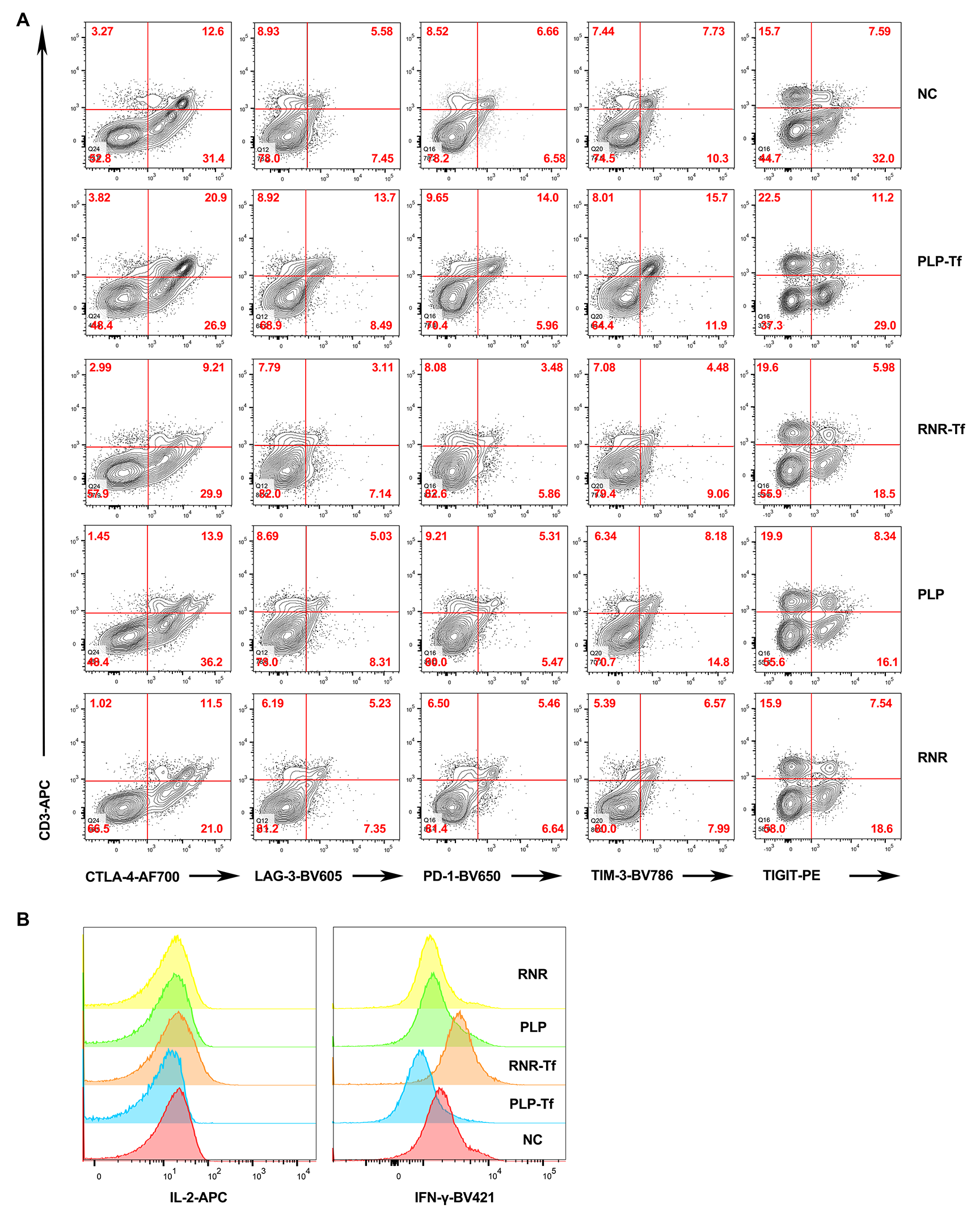

Supplement: Supplementary 1 — Supplementary Methods Figs. S1 to S12 Tables S1 and S2 Supplementary Reference [file research.0578.f1.zip › Fig S3.tif]

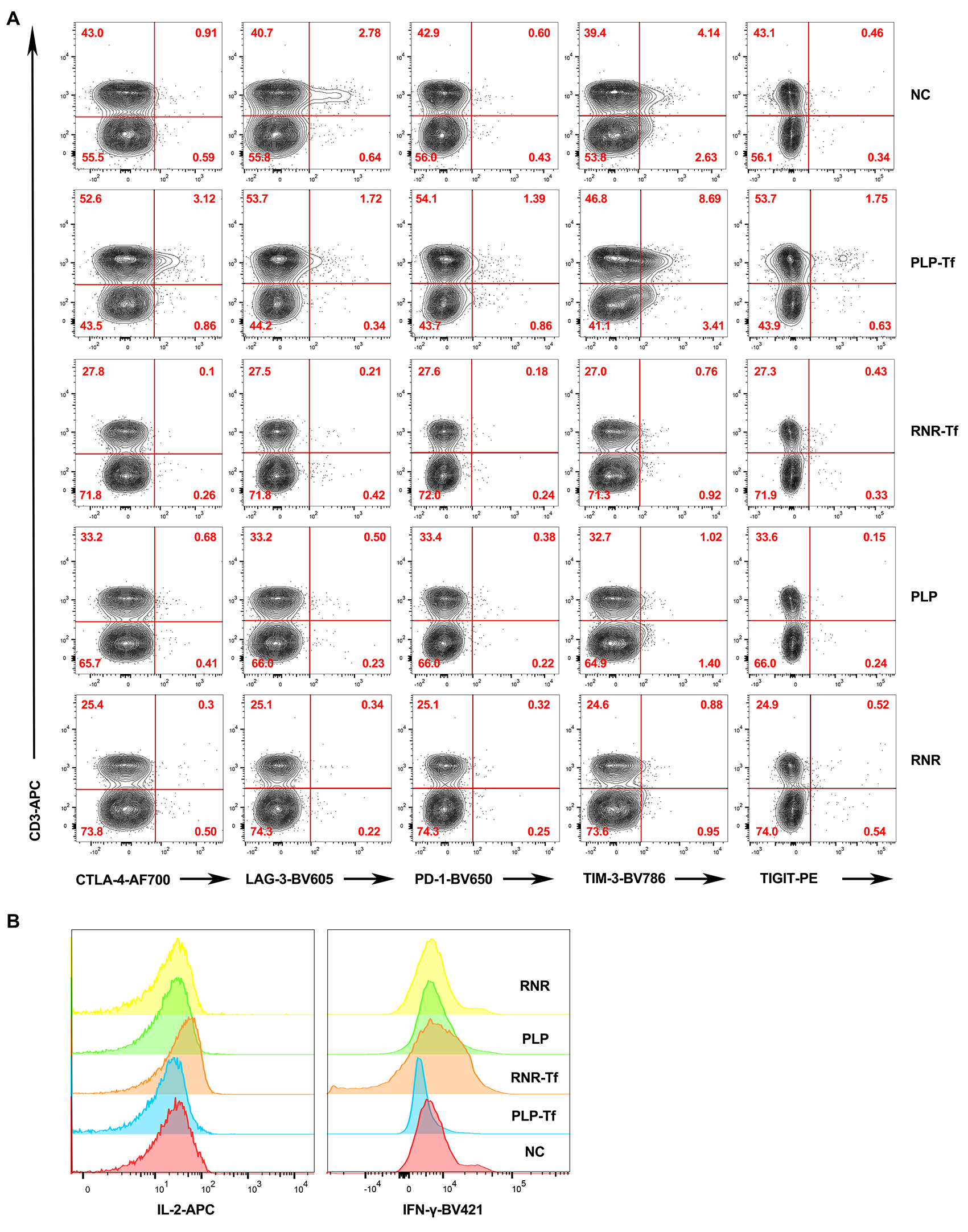

Supplement: Supplementary 1 — Supplementary Methods Figs. S1 to S12 Tables S1 and S2 Supplementary Reference [file research.0578.f1.zip › Fig S4.tif]

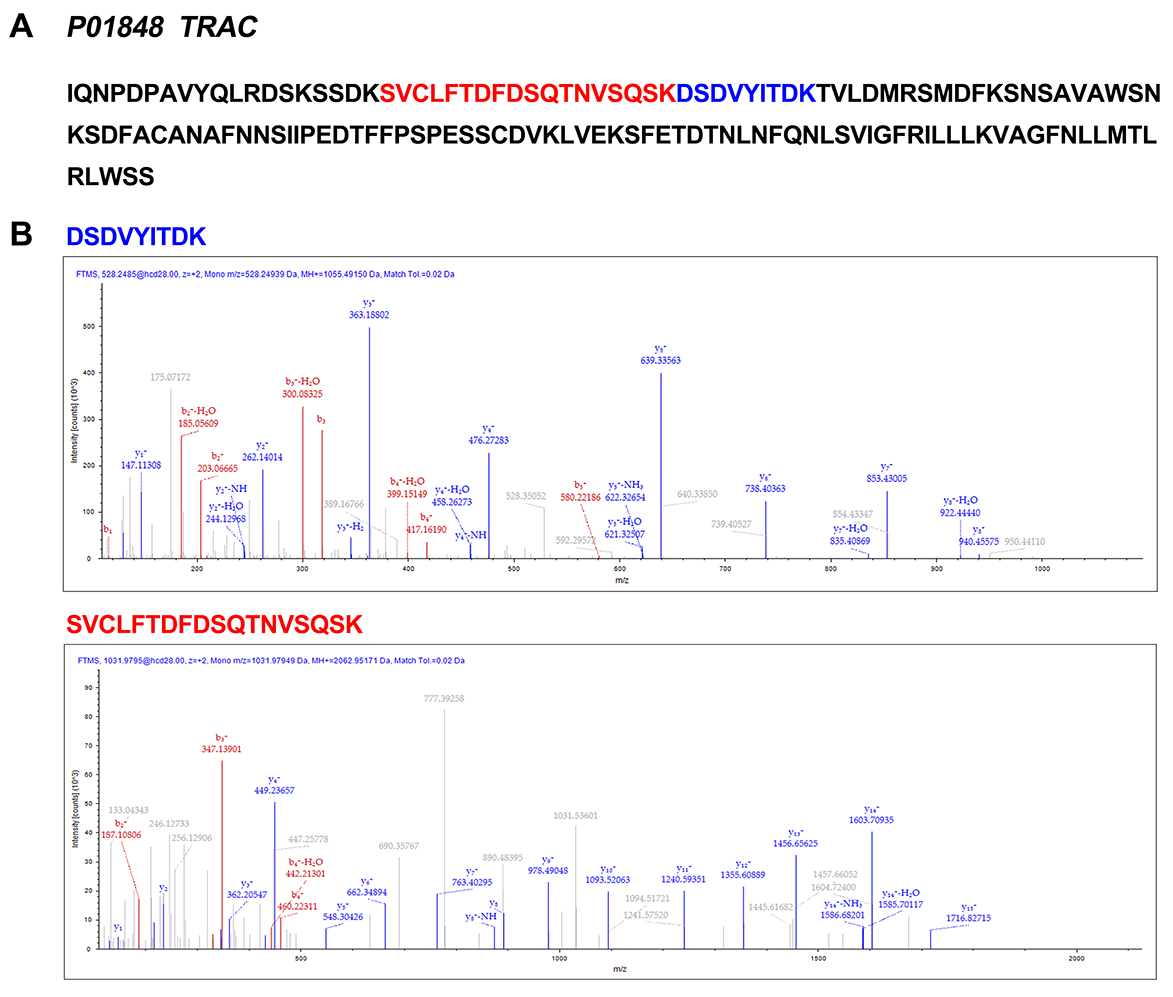

Supplement: Supplementary 1 — Supplementary Methods Figs. S1 to S12 Tables S1 and S2 Supplementary Reference [file research.0578.f1.zip › Fig S5.tif]

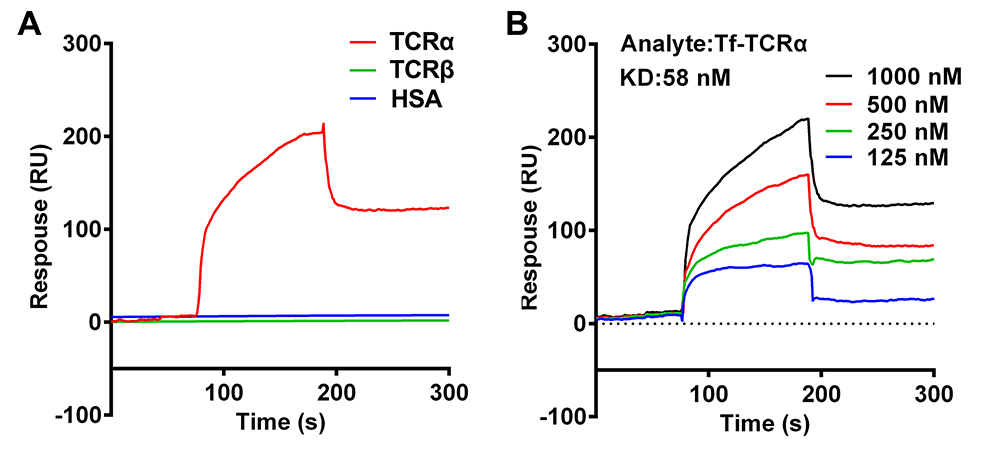

Supplement: Supplementary 1 — Supplementary Methods Figs. S1 to S12 Tables S1 and S2 Supplementary Reference [file research.0578.f1.zip › Fig S6.tif]

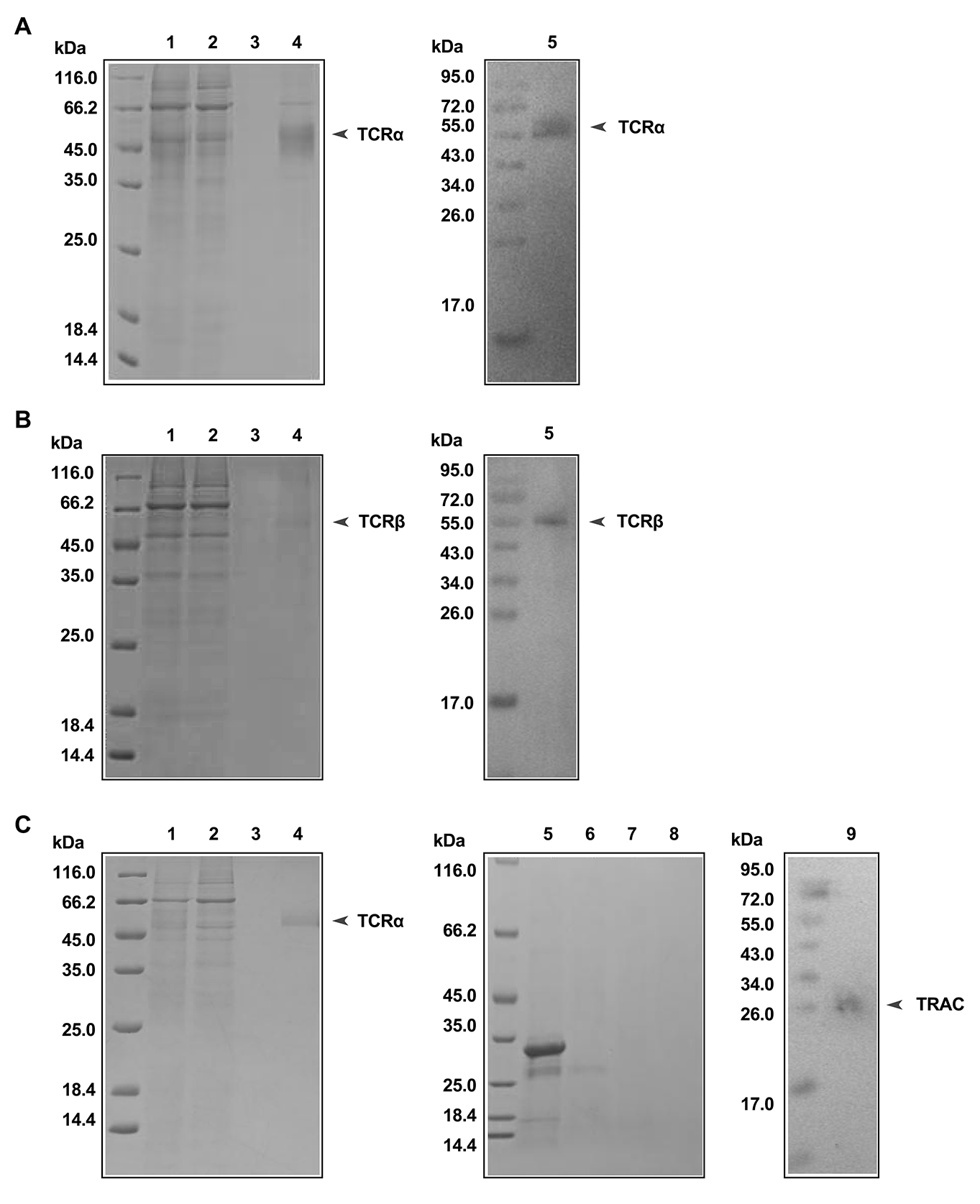

Supplement: Supplementary 1 — Supplementary Methods Figs. S1 to S12 Tables S1 and S2 Supplementary Reference [file research.0578.f1.zip › Fig S7.tif]

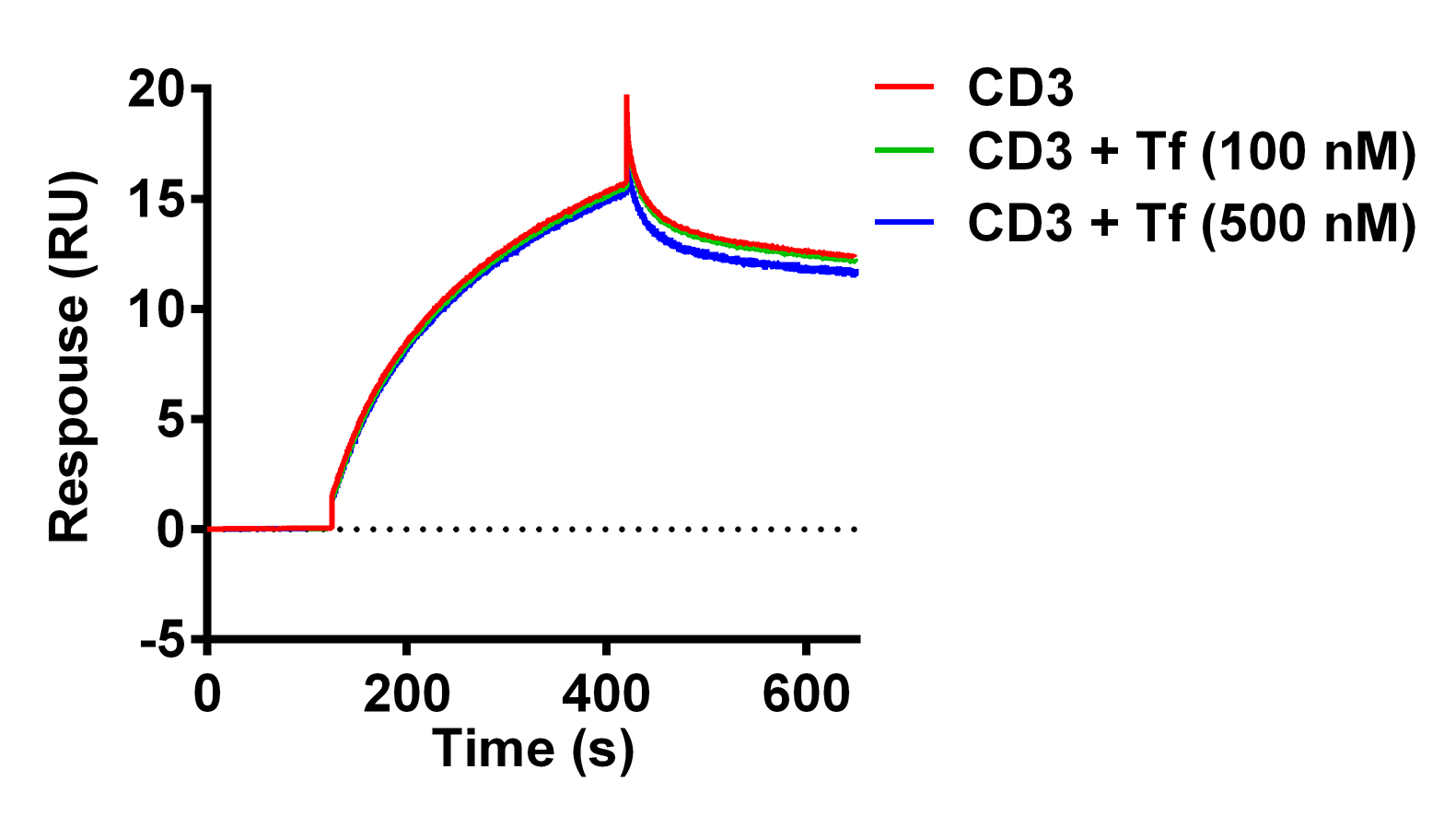

Supplement: Supplementary 1 — Supplementary Methods Figs. S1 to S12 Tables S1 and S2 Supplementary Reference [file research.0578.f1.zip › Fig S8.tif]

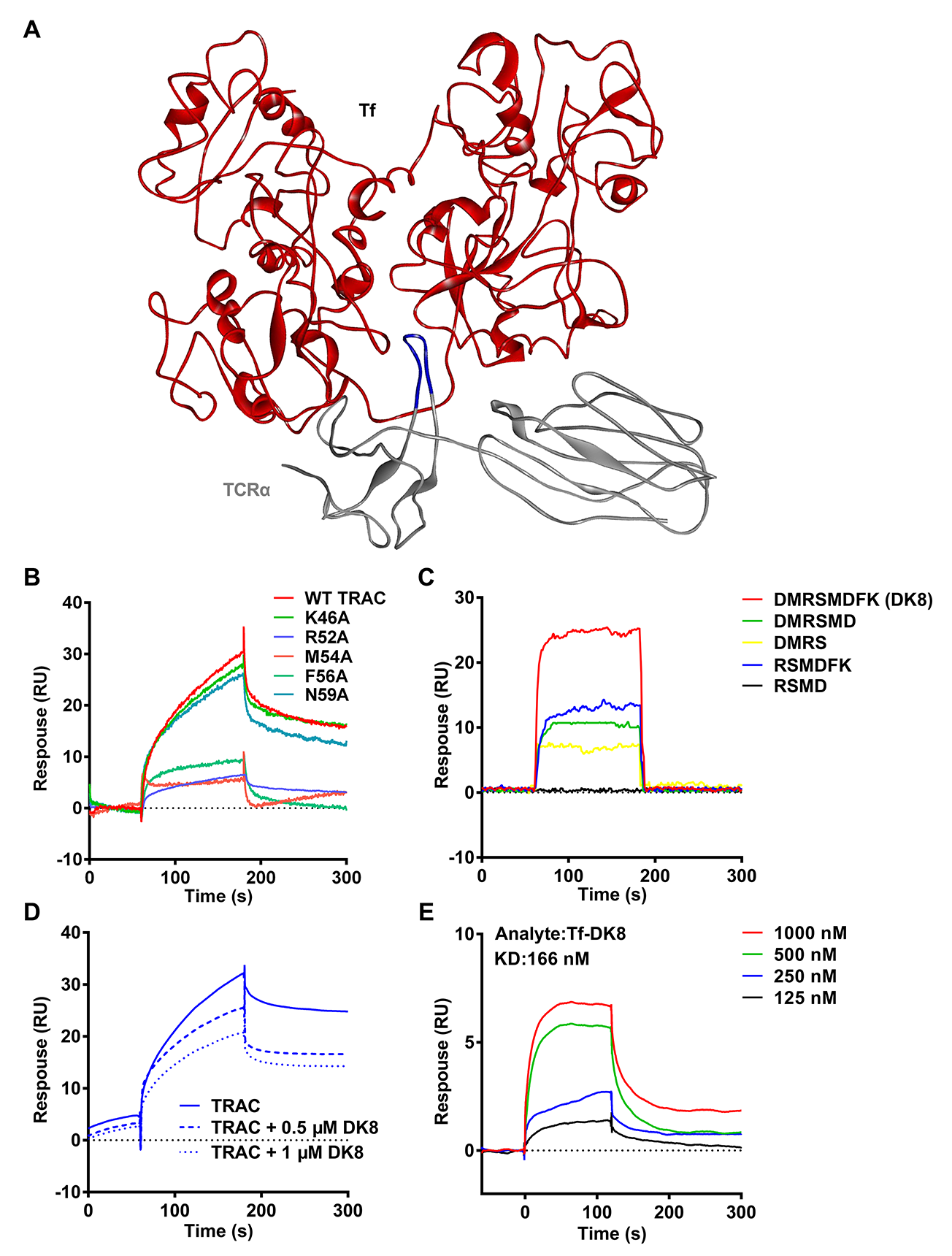

Supplement: Supplementary 1 — Supplementary Methods Figs. S1 to S12 Tables S1 and S2 Supplementary Reference [file research.0578.f1.zip › Fig S9.tif]
